# The Role of Reducibility of PtGaO*_x_*‐Based Catalysts for Efficient and Durable Propane Dehydrogenation

**DOI:** 10.1002/anie.202506704

**Published:** 2025-06-16

**Authors:** Kai Wu, Vita A. Kondratenko, Mingxia Zhou, Dmitry E. Doronkin, Stephan Bartling, Qiyang Zhang, Shanlei Han, Xin Jia, Qi Liu, Dong Xiong, Chunming Xu, Guiyuan Jiang, Dan Zhao, Uwe Rodemerck, David Linke, Evgenii V. Kondratenko

**Affiliations:** ^1^ State Key Laboratory of Heavy Oil Processing China University of Petroleum Beijing 102249 China; ^2^ Department: Advanced Methods for Applied Catalysis Leibniz‐Institut für Katalyse e. V Albert‐Einstein‐Str. 29a 18059 Rostock Germany; ^3^ Institute for Chemical Technology and Polymer Chemistry Institute of Catalysis Research and Technology Karlsruhe Institute of Technology Kaiserstr. 12 D‐76131 Karlsruhe Germany; ^4^ National Engineering Laboratory for Methanol to Olefins Dalian National Laboratory for Clean Energy iChEM (Collaborative Innovation Center of Chemistry for Energy Materials) Dalian Institute of Chemical Physics Chinese Academy of Sciences Dalian 116023 China

**Keywords:** Deactivation, Durability, GaO*_x_*‐based catalysts, Propane dehydrogenation, Reaction mechanism

## Abstract

Although PtGaO*_x_*‐containing catalysts are active and selective in the non‐oxidative dehydrogenation of propane (PDH) to propylene, they suffer from rapid deactivation and, especially, inability to recover their initial performance in a series of PDH/oxidative regeneration cycles, characteristics that are highly relevant to commercialization. Herein, we identified reducibility of GaO*_x_* as the key descriptor affecting the above catalyst features. Oxidized GaO_x_ species are more active than reduced GaO_x_ in the recombination of two H species formed from propane, which is the rate‐limiting step in the PDH reaction. This process is further accelerated by Pt. The reduction of GaO_x_ with time on propane stream leads to catalyst deactivation. Easily reducible GaO_x_ also tend to form PtGaO_x_ under PDH conditions, from which Pt atoms present in fresh catalysts cannot be completely recovered during oxidative regeneration, which is detrimental to catalyst durability. Regardless of the reaction atmosphere, Pt single atoms exist on the surface of PtGaO*_x_*‐containing catalysts with hardly reducible GaO_x_. Based on the knowledge derived, we developed a catalyst with 500 ppm Pt on the surface of mixed GaAlO*_x_*, which outperforms almost all previous PtGaO_x_‐containing catalysts in terms of space‐time yield of propylene formation and shows durable operation under industrially relevant conditions.

## Graphical abstract

The reaction‐induced reduction of oxidized GaO*_x_* was found to be the main cause of deactivation for PtGaO*_x_* catalysts in PDH because of the lower reactivity of reduced GaO*_x_* to form hydrogen through Pt‐assisted recombination of two surface H atoms formed from propane. This undesired transformation can be inhibited by increasing the strength of the Ga─O─Al bond. To control this property, GaAlO*_x_* solid solutions were used as supports for Pt and to avoid the presence of supported GaO*_x_* species.

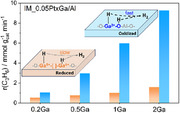

## Introduction

GaO*_x_*‐based catalysts have been the subject of intensive research in propane dehydrogenation (PDH) to propylene for years as possible alternatives to industrially used Pt‐ or CrO*_x_*‐based catalysts.^[^ ^1^, ^2^, ^3^ ^]^ Previous pioneering studies^[^ ^4^, ^5^, ^6^ ^]^ combined GaO*_x_* with Pt to produce active PDH catalysts. In the study of Sattler et al.,^[^ ^7^ ^]^ it was shown that even if Pt loading is as low as 0.1 wt%, the developed catalyst Pt3GaK/Al_2_O_3_ (3 wt% Ga, 0.25 wt% K) was highly active and selective. The PtGa‐Pb/SiO_2_ catalyst with atomically dispersed Pt reported in Ref. [8] performed superior to commercially relevant Pt‐Sn‐containing catalysts at the same amount of exposed Pt. Thus, the Fluidized Catalytic Dehydrogenation process developed by Dow utilizes a Pt‐Ga‐K/Si‐Al_2_O_3_ catalyst.^[^ ^9^ ^]^

Almost all (Pt)Ga‐containing catalysts, that have been reported so far, deactivate with time on propane stream in the absence of co‐fed H_2_.^[^ ^10^ ^]^ Moreover, they are unable to restore their high initial activity in a series of dehydrogenation/oxidative regeneration cycles. For example, the Pt3GaK/Al_2_O_3_ catalyst,^[^ ^7^ ^]^ one of the most promising materials, showed an irreversible decrease in propane conversion and propylene selectivity from about 46% to about 31% and from about 96% to about 92.5% in the first 10 dehydrogenation/regeneration cycles, respectively. The catalyst began to perform durable after 2 days. The deactivation behavior during the first 10 cycles was not explained although those authors found that the dispersion of Pt increased after catalyst exposure to air at 650 °C. The resistance of Pt against sintering could be improved by increasing the strength of Pt interaction with the support as for example shown by Zhu and co‐workers^[^ ^11^ ^]^ who reported that pentacoordinated Al^3+^ (Al^3+^ penta) strongly anchors Pt. The activity of the developed Pt‐Sn/Al_2_O_3_ catalyst with 50.8% of Al^3+^ penta sites could be fully restored after oxidative regeneration. This approach was, however, ineffective when Ga was used instead of Sn.^[^ ^12^ ^]^ The introduction of Ce to a Pt‐Ga/Al system could improve the catalyst durability.^[^ ^13^, ^14^ ^]^ Owing to strong Pt─O─Ce interactions, the particle size of Pt clusters on the surface of 1Ce‐PtGa/Al_2_O_3_ (1 wt% Ce) increased from 1.1 to 4.4 nm after 20 PDH/regeneration cycles, while the corresponding changes in the case of its Ce‐free counterpart were from 1.1 to 18 nm. Through regulating the Ce content in Ce‐PtGa/Al_2_O_3_ catalysts, Choi and co‐workers^[^ ^15^ ^]^ revealed that 2 wt% of Ce is an optimal content to perform durable.

In addition to the uncertainties discussed above regarding the mechanism of catalyst deactivation and durability, there are also debates regarding the nature of the active sites in supported Pt‐Ga‐containing catalysts. Jablonski et al.,^[^ ^4^ ^]^ who first introduced PtGa/Al_2_O_3_ catalysts, suggested that the role of Ga is to dilute catalytically active Pt sites. Instead, Weckhuysen and co‐workers concluded that coordinatively unsaturated Ga^3+^ sites are the active species, while Pt present in trace amounts (0.1 wt%) acts as a promoter accelerating the recombination of the hydrogen atoms formed from propane.^[^ ^7^ ^]^ A similar conclusion has also been made by Choi and co‐workers, who further pointed out that Pt^0^ species facilitate the recombination of surface H species via reverse spillover while Pt^2+^ is inactive in this process. In addition, PtGa alloy^[^ ^16^, ^17^, ^18^ ^]^ and intermetallic^[^ ^8^ ^]^ species were considered as active species. Recently, Gong and co‐workers^[^ ^19^ ^]^ investigated Pt‐modified Ga_2_O_3_‐based catalysts and established a linear relationship between the rate of propylene formation and Ga^δ+^‐H species. The latter were suggested to be the key species for PDH, while the role of Pt is to facilitate their formation. This synergistic effect, however, is diminished by the formation of PtGa alloys when Pt loading exceeds 0.1 wt%. The key role of the metastable gallium hydride species was later supported by the same group using a series of Al_2_O_3_‐supported Ga‐containing catalysts.^[^ ^20^ ^]^

Finally, the kind of pretreatment of (Pt)Ga‐based catalysts before starting PDH has a crucial effect on their activity. For example, a Ga/SiO_2_ catalyst treated in He or H_2_ at 550 °C exhibited similar catalytic performance.^[^ ^21^ ^]^ However the conversion decreased when the catalyst was reduced at 650 °C due to the loss of tetracoordinate Ga^3+^‐O sites. In the studies, in which Pt─Ga alloys are deemed as active species, the catalysts were reduced before PDH, although it is known that non‐treated PtGa‐based catalysts also exhibit considerable PDH activity.^[^ ^7^, ^12^, ^15^ ^]^

Motivated by the industrial significance of the PtGa/Al_2_O_3_ system and the discrepancies in the fundamentals regarding the kind of active sites, their formation and, especially, the origins of catalyst stability/durability, the present study was performed to close this gap. To this end, we prepared a series of catalysts differing in the ratio of Pt/Ga and in the redox properties of Ga‐containing species/phases. Catalytic tests with reduced or oxidized catalysts and with in situ titration of reduced GaO*_x_* species using O_2_ helped us to conclude that oxidized GaO_x_ species are more active and, in particular, more selective in the PDH reaction compared to their reduced counterparts. Temporal analysis of products and density functional theory calculations suggest that the formation of H_2_ through the recombination of atomically adsorbed hydrogen species is the rate limiting step in this reaction. This step is accelerated by Pt species and occurs easier on oxidized GaO*_x_* species. The fundamentals elucidated in this study were instrumental to develop PtGa/Al_2_O_3_ catalysts, which totally restore their initial performance in a series of dehydrogenation/regeneration cycles in the temperature range between 550 °C and 625 °C in the absence or presence of H_2_. The regeneration was simply performed at the reaction temperatures using air.

## Results and Discussion

### Active Species and Deactivation Mechanism of Pt‐GaO*_x_*/Al_2_O_3_ Catalysts in PDH

**Figure 1:**
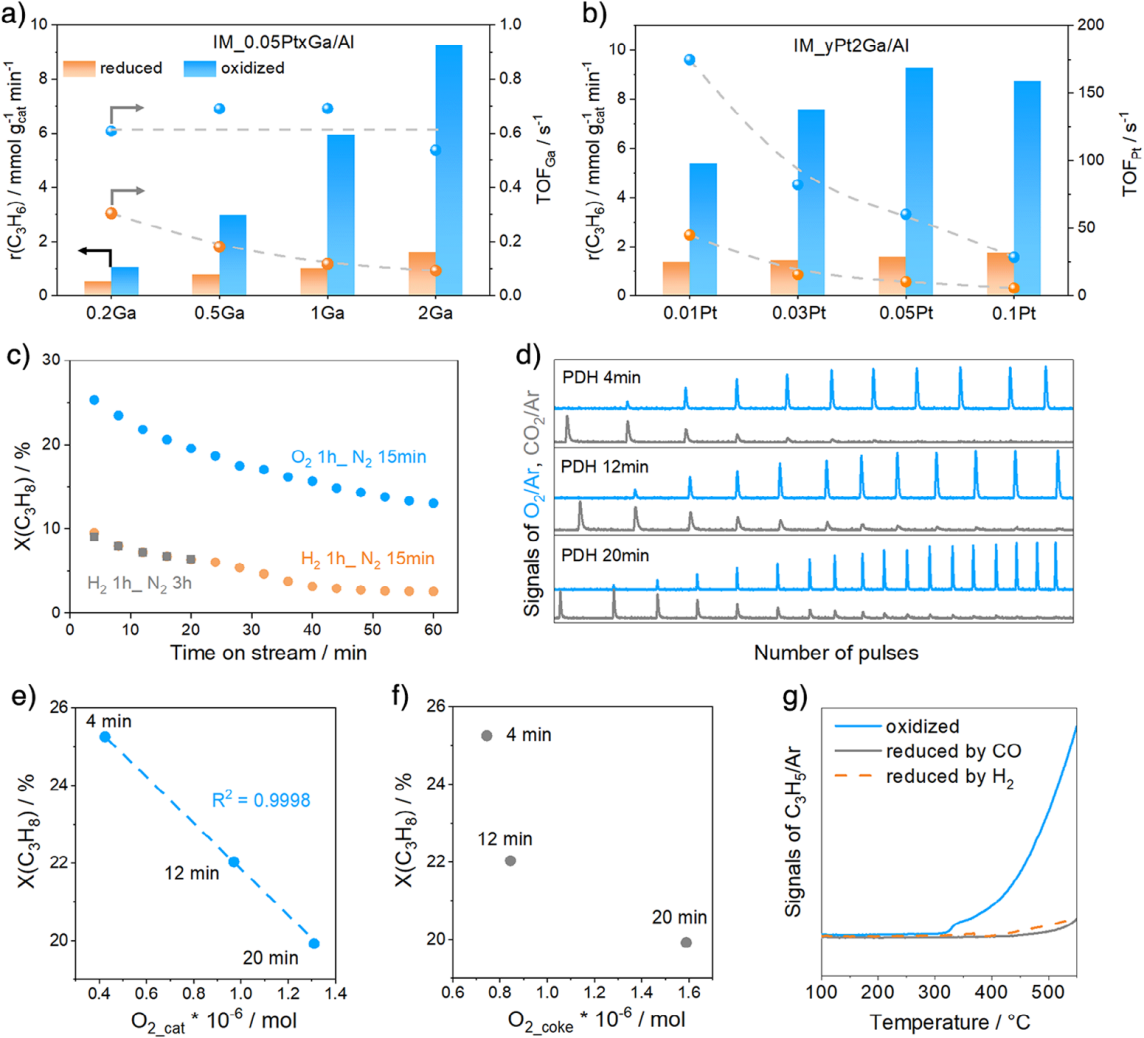
a) The rate (r(C_3_H_6_)) and Ga‐related turnover frequency (TOF_Ga_) of propylene formation over IM_0.05PtxGa/Al versus Ga loading. b) The r(C_3_H_6_) and Pt‐related TOF (TOF_Pt_) over IM_yPt2Ga/Al versus Pt loading. c) Propane conversion (X(C_3_H_8_)) after different N_2_ purge durations in PDH over oxidized or reduced catalysts. d) MS signals of O_2_ and CO_2_ during multiple pulses of a 1 vol% O_2_/He mixture after different times on propane stream. e)–f) Relationships between X(C_3_H_8_) and the amount of O_2_ consumed for reoxidation of reduced GaO*_x_* (O_2_cat_) or coke oxidation (O_2_coke_). For calculations details see Figure S2 and the corresponding description. g) MS signals of C_3_H_5_/Ar during C_3_H_8_‐TPSR over oxidized, CO‐reduced, or H_2_‐reduced catalysts. PDH reaction conditions for a) and b): 550 °C, C_3_H_8_/N_2_ = 2/3, *m* = 0.01–0.05 g, *F*_total_ = 40–70 mL min^−1^; PDH reaction conditions for c): 550 °C, C_3_H_8_/N_2 _= 2/3, *m* = 0.05 g, WHSV(C_3_H_8_) = 37.7 h^−1^.

To understand the role of Pt and GaO*_x_* in the Pt‐GaO*_x_*/Al_2_O_3_ catalysts, two series of materials with a fixed Pt content of only 0.05 wt% but different Ga loadings (IM_0.05Pt*x*Ga/Al, 0.2 ≤ *x* ≤ 2 wt%) or with a fixed loading of Ga of 2 wt% but different contents of Pt (IM_*y*Pt2Ga/Al, 0.01 ≤ *y* ≤ 0.1 wt%) were synthesized by an incipient wetness method. The catalysts were either reduced or oxidized before PDH tests performed under kinetically controlled conditions. The rate of propylene formation determined over the IM_0.05Pt*x*Ga/Al catalysts at 550 °C increased with an increase in Ga loading irrespective of the type of catalyst treatment (Figure 1a). Importantly, the oxidized samples showed higher activity in comparison to their reductively treated counterparts. This is also valid for the IM_*y*Pt2Ga/Al catalysts (Figure 1b). The rate over the latter only slightly increased with increasing Pt loading.

We also calculated the turnover frequency of propylene formation with respect to Ga (TOF_Ga_) or Pt (TOF_Pt_) under assumption that each Ga or Pt atom catalyzes PDH (Figure 1a,b). For the oxidatively treated IM_0.05Pt*x*Ga/Al catalysts, their TOF_Ga_ values are close to each other (about 0.6 s^−1^). However, for their reductively treated counterparts, this value decreases from 0.3 to 0.1 s^−1^ with increasing Ga loading (Figure 1a). Regardless of the type of treatment of the IM_*y*Pt2Ga/Al catalysts, TOF_Pt_ decreases with increasing Pt loading (Figure 1b). In addition, considering that the propane conversion over IM_0.05Pt/Al is only 1%, which is three times lower than that obtained over IM_2Ga/Al, Pt should not be the active species (Figure S1). Since the TOF_Ga_ value of the oxidatively treated IM_0.05Pt*x*Ga/Al catalysts is independent of the Ga loading, we put forward that oxidized GaO*_x_* species should be actively involved in PDH. The different TOF_Ga_ values of the reduced IM_PtGa/Al catalysts should be related to the different concentrations of oxidized GaO*_x_* as the catalysts differ in their reducibility (see the related discussion in **verifying the role of reducibility of GaO*_x_* for catalyst activity**).

The conversion of propane over oxidized or reduced IM_0.05Pt2Ga/Al decreased from 25% to 13% or from 9.5% to 2.5% within 60 min on propane stream (Figure 1c). To check if the formation of coke is the only dominant reason for the deactivation in the course of PDH, as generally postulated for GaO*_x_*‐based catalysts,^[^ ^22^ ^]^ we performed additional PDH tests using oxidized catalysts as follows. The reaction was stopped after different times on propane stream (4, 12, or 20 min) followed by pulsing a 1 vol% O_2_/He mixture until no obvious O_2_ consumption was observed (Figures 1d and S2). The O_2_ in the first pulse was completely consumed resulting in the formation of CO_2_. Then, the peak areas of O_2_ and CO_2_ did not change after 7, 9, or 15 O_2_ pulses in the tests with the catalysts reacting with C_3_H_8_ for 4, 12, or 20 min, indicating that the oxidation of carbon‐containing deposits was complete. O_2_ could be consumed for both oxidation of carbon‐containing surface deposits and reoxidation of reduced GaO*_x_* sites. To distinguish these processes, we evaluated the O_2_ pulse tests as follows. The amount of O_2_ consumed for coke combustion (O_2_coke_) was assumed to be equal to the amount of CO_2_ formed. The amount of O_2_ consumed (O_2_cat_) for the reoxidation of reduced GaO*_x_* species is equal to the difference between the total amount of O_2_ consumed (O_2_total_) and O_2_coke_. The O_2_cat_ amount was determined to be 4.2 × 10^−7^, 9.7 × 10^−7^, or 1.3 × 10^−6^ mol over the catalyst exposed to propane stream after 4, 12, or 20 min (Figure S2). This amount correlates linearly with X(C_3_H_8_) determined after the same times on propane stream (Figure 1e). No correlations between X(C_3_H_8_) and the O_2_coke_ amount could be found (Figure 1f). The latter result suggests that the formation of coke cannot be the dominant reason for catalyst deactivation with increasing time on propane stream.

Considering the higher activity of the oxidized IM_0.05Pt*x*Ga/Al catalysts in comparison with their reduced counterparts, as well as the correlation in Figure 1e, we put forward that the reduction of oxidized GaO*_x_* species also contributes to catalyst deactivation in the course of PDH. The formation of Ga^δ+^‐H can be excluded to cause the deactivation because H_2_‐ or CO‐reduced catalysts did not differ in their activity to dehydrogenate C_3_H_8_ to C_3_H_6_ (Figure 1g). Ga^δ+^‐H does not seem to exist on the surface of the CO‐reduced catalysts. Besides, no change in catalyst activity was observed when prolonging purging time in N_2_ from 15 min to 3 h (Figure 1c). Ga^δ+^‐H species are known to be unstable in an inert atmosphere.^[^ ^19^ ^]^ Therefore, Ga^δ+^O*_x_* with an oxygen vacancy and/or Ga^0^ species formed under reaction conditions should show lower activity than oxidized Ga^3+^O*_x_*.

### Verifying the Role of Reducibility of GaO*_x_* for Catalyst Activity

**Figure 2:**
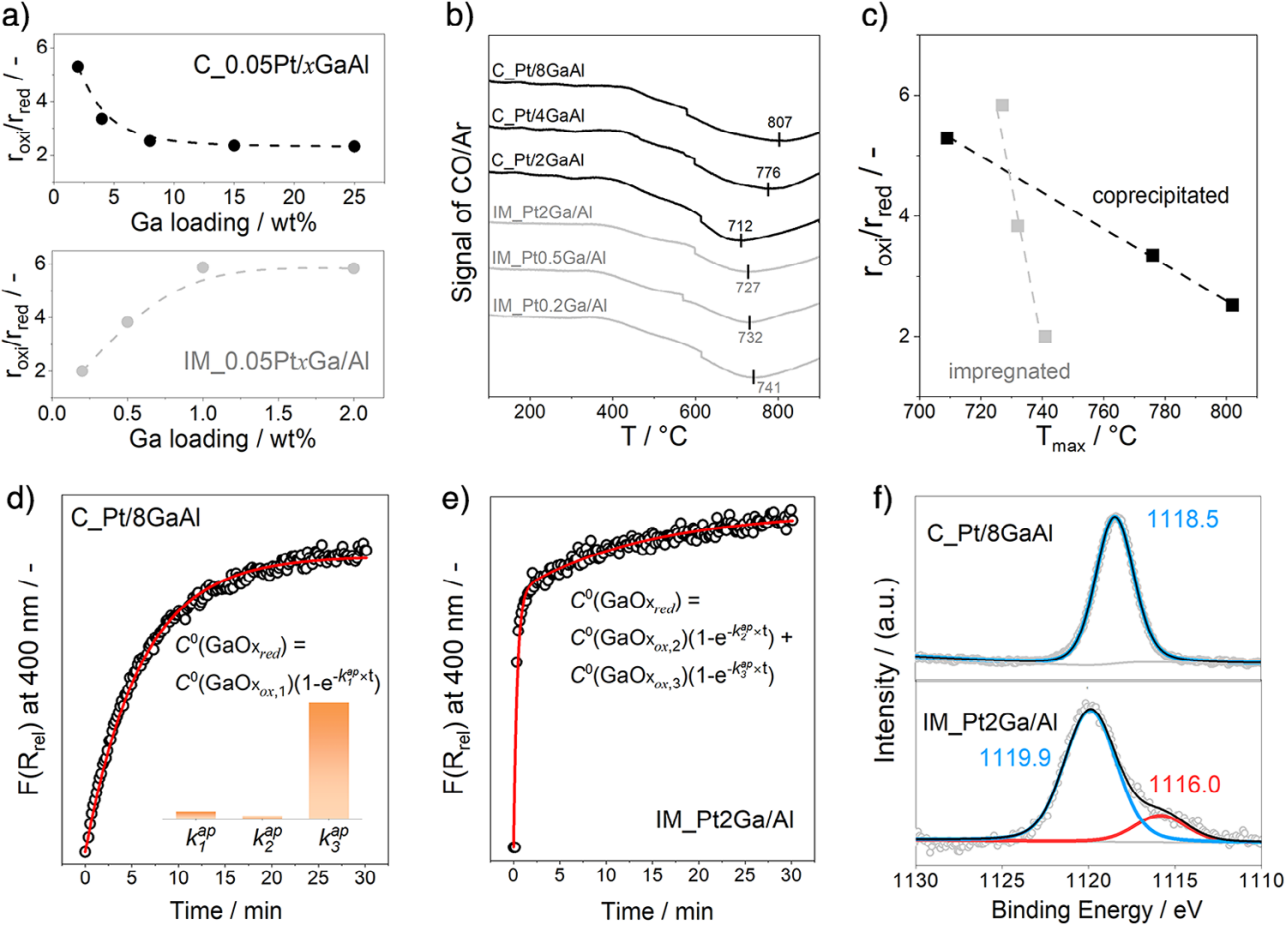
a) The effect of Ga loading on the ratio of the rate of propylene formation over oxidized C_Pt/xGaAl (top) or IM_PtxGa/Al (bottom) to that over their reduced counterparts (r_oxi_/r_red_). b) CO‐TPR profiles of C_Pt/xGaAl (black line) and IM_PtxGa/Al (gray line). c) A correlation between the r_oxi_/r_red_ ratio and the temperature of maximum CO consumption (*T*_max_). d)–e) Temporal changes (circles) in the Kubelka–Munk function at 400 nm during reduction of oxidized C_Pt/8GaAl and IM_Pt2Ga/Al by H_2_, the fit (red line) to the models shown in d) and e) and the apparent rate constants of the reduction of these catalysts as obtained from the fit (d) inset). f) Ga 2p_3/2_ XP spectra of oxidized C_Pt/8GaAl and IM_Pt2Ga/Al.

To check experimentally if the extent of catalyst deactivation can be controlled by the reducibility of GaO*_x_* species, we prepared a series of catalysts based on a GaAlO*_x_* solid solution used as a support for Pt species. To distinguish them from the above‐mentioned catalysts, we use the abbreviation C_Pt/GaAl. Consistent with the IM_PtGa/Al catalysts, the oxidized C_Pt/GaAl catalysts showed higher activity than their reduced counterparts (Figure S3). However, the differently prepared catalysts differ in the effect of Ga loading on the rate of propylene formation over oxidized (r_oxi_) and reduced (r_red_) catalysts. We used the r_oxi_/r_red_ ratio to denote the decrease in the rate of propene formation over reductively treated catalysts in comparison with their oxidatively treated counterparts. The r_oxi_/r_red_ ratio over C_Pt/GaAl decreases from 5.3 to 2.3 with an increase in the loading from 2 to 25 wt% (Figure 2a). The decrease is due to the increase in the r_red_, while the r_oxi_ was not affected (Figure S3). On the contrary, this ratio increases from 2 to about 6 over IM_PtGa/Al with rising loading from 0.2 to 2 wt% because of the increase in the r_oxi_ (Figures 1a and 2a).

CO‐TPR tests revealed that the C_Pt/GaAl and IM_PtGa/Al catalysts consume CO in a broad temperature range (Figure 2b). The Ga loading in the C_Pt/GaAl catalysts negatively affects the reduction of GaO*_x_* species as concluded from the increase in the temperature of the maximum of CO consumption (*T*_max_). The *T*_max_ values of C_Pt/2GaAl, C_Pt/4GaAl, and C_Pt/8GaAl are 712, 776, and 807 °C, respectively. The *T*_max_ of the IM_PtGa/Al catalysts, however, shifts to lower temperatures as the Ga loading increases. For both catalyst types, the higher the *T*_max_ value, the lower the r_oxi_/r_red_ ratio (Figure 2c). As oxidized catalysts show higher PDH activity than their reduced counterparts, the correlation between the r_oxi_/r_red_ and *T*_max_ values suggests that the inhibition of the reducibility of PtGaO*_x_*‐based catalysts can be effective in improving the catalytic activity.

Inspired by previous studies dealing with VO*_x_*‐based catalysts,^[^ ^23^ ^]^ the reduction kinetics of oxidized GaO*_x_* species at 550 °C using a 25 vol% H_2_/N_2_ feed was analyzed by time‐resolved in situ UV–vis spectroscopy. Since the intensity of the Kubelka–Munk function at around 400 nm increased with rising time on H_2_ stream (Figure S4), we consider the relative Kubelka–Munk function (F(R_rel_)) at this wavelength for analyzing the kinetics of the reduction of oxidized GaO*_x_*. Figure 2d,e shows the temporal changes in the F(R_rel_) of C_Pt/8GaAl and IM_Pt2Ga/Al. For both oxidized catalysts, the F(R_rel_) increased from 0 (there are no reduced GaO*_x_* sites in the oxidized catalysts) after we switched from an O_2_‐containing flow (20 vol% O_2_/N_2_) to a H_2_‐containing flow (25 vol% H_2_/N_2_). The obtained time‐resolved profiles of F(R_rel_) were fitted to a simple kinetic model shown in Equation 1 (see the Supporting Information for details).

(1) $$C\;\left(\mathrm{Ga}O_{x_{red}}\right)=C^{0}\;\left(\mathrm{Ga}O_{x_{ox,1}}\right)\times \left(1-e^{-k_{1}^{ap}\times t}\right)$$

where $k_{1}^{ap}$ is the apparent rate constant (*k*_1_ × *p*(*H*_2_)) of the reduction of oxidized GaO*_x_* species, $C(\mathrm{Ga}O_{x_{red}})$ is the concentration of reduced GaO*_x_* species, while $C^{0}\left(\mathrm{Ga}O_{x_{ox,1}}\right)$ is the initial concentration of oxidized GaO*_x_*.

It is obvious that this model describes the experimental data obtained with the C_Pt/8GaAl catalyst correctly (Figure 2d) but failed for the IM_Pt2Ga/Al catalyst (Figure S5). Thus, we suggested that at least two differently reducible GaO*_x_* species should be present on the surface of the IM_Pt2Ga/Al catalyst. The formation of reduced GaO_x_ species in this case can be described by Equation 2.

(2) $$\begin{array}{ccc} C\;\left(\mathrm{Ga}O_{x_{red}}\right) & = & C^{0}\;\left(\mathrm{Ga}O_{x_{ox,2}}\right)\times \left(1-e^{-k_{2}^{ap}\times t}\right)+C^{0}\left(\mathrm{Ga}O_{x_{ox,3}}\right)\\ & & \times \;(1-e^{-k_{3}^{ap}\times t}) \end{array}$$

where $k_{2}^{ap}$ and $k_{3}^{ap}$ are the apparent rate constants (*k*_2_ × *p*(*H*_2_) and *k*_3_ × *p*(*H*_2_)) of the reduction of oxidized GaO*_x_*_,2_ and GaO*_x,_*_3_ species, respectively. Their initial concentration is abbreviated as $C^{0}\left(\mathrm{Ga}O_{x_{ox,2}}\right)$ and $C^{0}\left(\mathrm{Ga}O_{x_{ox,3}}\right)$.

This dual‐site model describes the temporal F(R_rel_) profiles recorded during the reduction of the IM_Pt2Ga/Al catalyst correctly. The obtained kinetic parameters of both catalysts are summarized in the insert of Figure 2d. It is also worth mentioning that the $k_{3}^{ap}$ value is much greater than the $k_{1}^{ap}$ and $k_{2}^{ap}$ values, which do not obviously differ from each other. This means that GaO*_x_* species characterized by $k_{3}^{ap}$ are more reactive towards reduction by H_2_ than those characterized by $k_{1}^{ap}$ or $k_{2}^{ap}$. Based on the below analysis, we suggest that Ga_2_O_3_ and Ga─O─Al sites should be easily and heavily reducible species, respectively.

X‐ray photoelectron (XP) spectroscopy was applied to determine the chemical state of gallium on the surface of C_Pt/8GaAl and IM_Pt2Ga/Al (Figure 2f). The binding energy at 1118.5 eV in the Ga 2p_3/2_ XP spectrum of the C_Pt/8GaAl catalyst can be ascribed to Ga^3+^.^[^ ^24^ ^]^ The Ga 2p_3/2_ XP spectrum of the IM_Pt2Ga/Al catalyst is characterized by the maximum at 1119.9 eV, which is still ascribed to Ga^3+^ species.^[^ ^19^ ^]^ A shoulder at 1116.0 eV could also be identified through deconvolution. The difference in the main binding energies between these catalysts was also present in the corresponding Pt‐free samples (Figure S6) and should be related to the presence of different GaO*_x_* species, which is consistent with the UV–vis analysis in Figure 2e. The binding energy of 74.5 eV in the Al 2p XP spectrum of the IM_2Ga/Al sample is typical for alumina^[^ ^25^ ^]^ and is slightly higher than 74.2 eV in the spectrum of the C_2GaAl sample (Figure S7). As Ga has higher electronegativity than Al (1.81 versus 1.61), the charge transfer contribution changes with the formation of Ga─O─Al linkages.^[^ ^26^ ^]^ Thus, the slightly lower Al 2p binding energy indicates the presence of Ga─O─Al motifs in the C_2GaAl sample. According to Equation 2, the parameters $C^{0}\left(\mathrm{Ga}O_{x_{ox,2}}\right)$ and $C^{0}\left(\mathrm{Ga}O_{x_{ox,3}}\right)$ represent the concentration of two different types of oxidized GaO*_x_* species. The ratio of these parameters is 5/1.4, which is similar to the ratio (5/1) of the peak areas of the Ga 2p_3/2_ XP signals at 1119.9 and 1116.0 eV.

Insights into the local structure of GaO_x_ species in the C_0.05Pt/8GaAl and IM_0.05Pt2Ga/Al samples were derived from X‐ray absorption spectroscopy (XAS) analysis. The obtained XANES and EXAFS spectra are given in Figures S8 and S9. In both samples, the oxidation state of Ga should be 3^+^ as evidenced by the absorption edge and the intensity of the white line (Figure S8a). From the EXAFS fitting results (Table S2), there are two different GaO*_x_* species in each catalyst but with different coordination numbers and Ga─(O)─Ga and Ga─(O)─Al distances (Figure S8b). In comparison with IM_0.05Pt2Ga/Al sample, higher coordination numbers of Ga─O─Ga and Ga─O─Al were obtained in C_0.05Pt/8GaAl, indicating that the stronger interaction of GaO_x_ species with AlO_x_ material.

### Products Selectivity and Coke Formation

**Figure 3:**
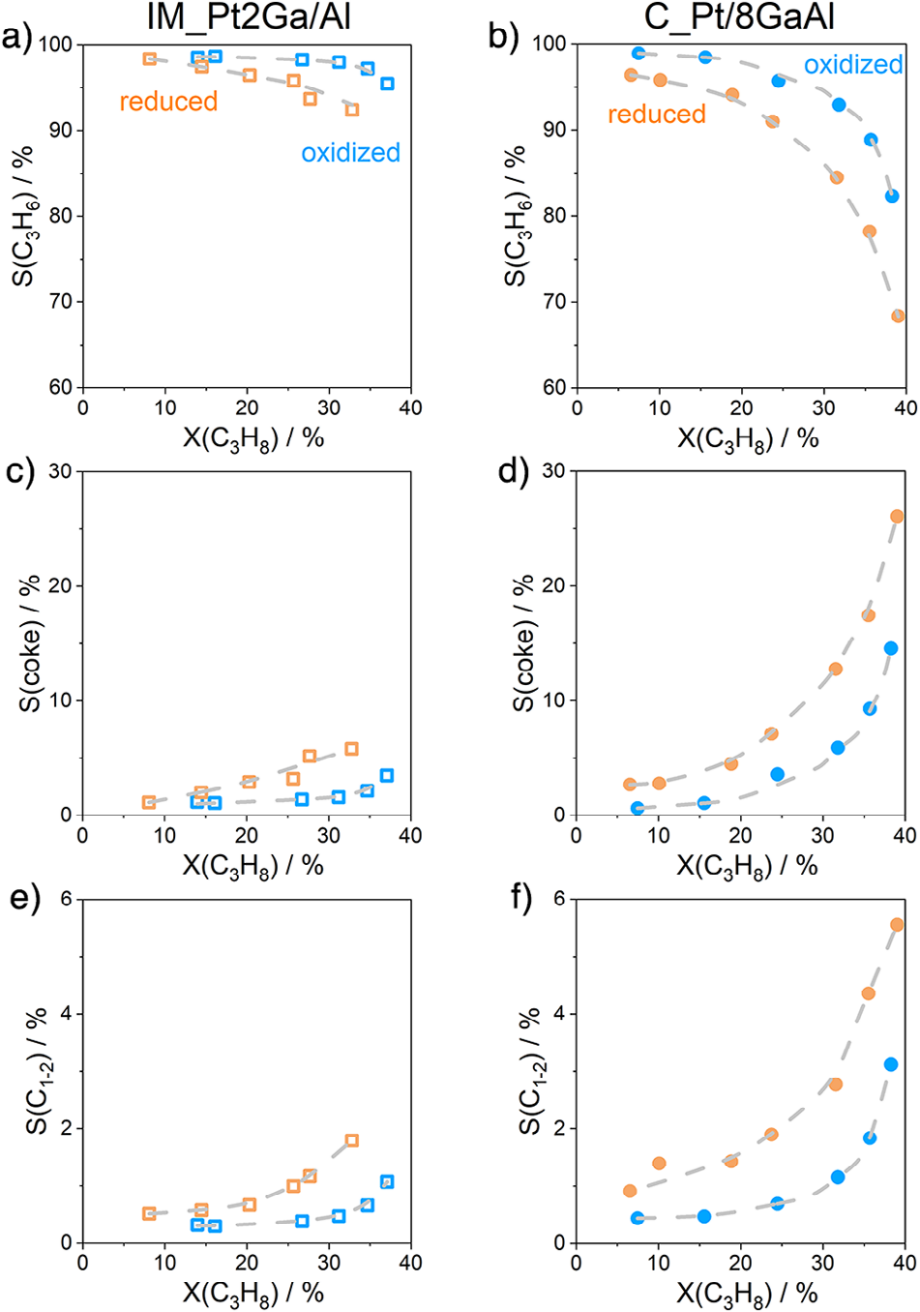
Selectivity‐conversion relationships for a), b) propylene, c), d) coke, and e), f) C_1‐2_ hydrocarbons over differently treated a), c), and e) IM_Pt2Ga/Al and b), d), and f) C_Pt/8GaAl.

Irrespective of the type of catalyst and its treatment before PDH, the selectivity to propylene decreased but the selectivity to cracking products (C_1‐2_ hydrocarbons) and coke increased when propane conversion increased (Figure 3). The selectivity to these products extrapolated to a zero degree of propane conversion is about 100%, 0%, and 0%, respectively. Thus, propylene seems to be the only primary product formed from propane, while coke and cracking products are formed by the consecutive reactions of propylene. However, the decrease in propylene selectivity with increasing propane conversion is more pronounced for reduced catalysts because of their enhanced ability for consecutive propylene transformations (Figure 3). In addition, C_Pt/8GaAl shows higher reactivity in the conversion of propylene to coke and cracking products in comparison with IM_Pt2Ga/Al. Catalyst acidity that is often considered to affect coke selectivity in the PDH reaction appears to influence coke formation over the present catalysts (Figure S10).

Based on the above discussion, it is counterintuitive that the C_Pt/8GaAl catalyst with higher ability to form coke showed higher on‐stream stability than the IM_Pt2Ga/Al catalyst (Figure S11). Therefore, the only explanation is that coke deposition is not the only cause of catalyst deactivation. As reduced catalysts are less active than their oxidized counterparts, we put forward that in situ reduction of oxidized GaO*_x_* species is another factor affecting catalyst on‐stream stability.

### Mechanistic Origins of Different Reactivity of Oxidized or Partially Reduced Pt‐GaO*_x_*‐Based Catalysts in PDH

**Figure 4:**
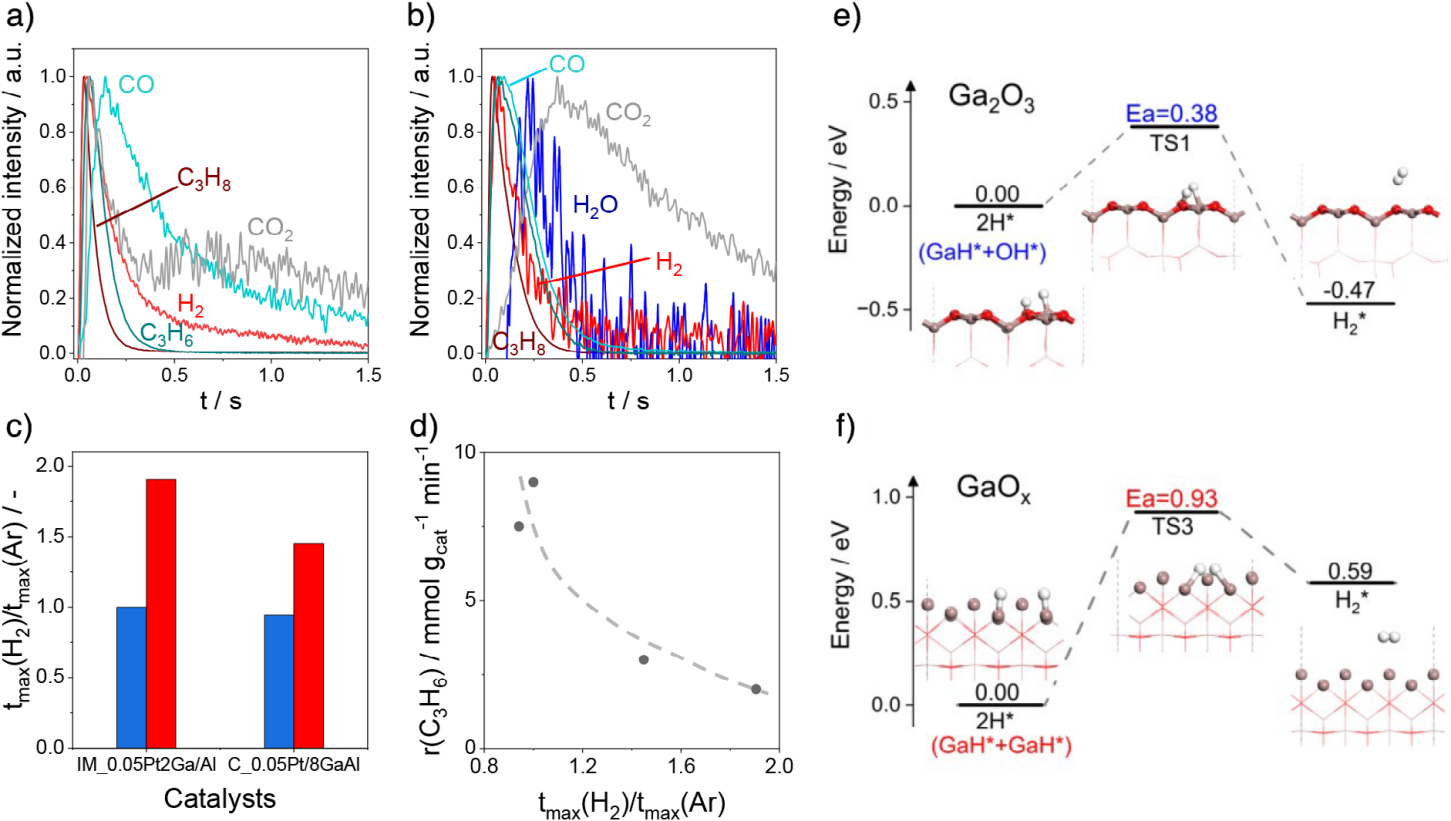
Normalized transient responses of C_3_H_8_ (brown), C_3_H_6_ (turquoise), CO_2_ (grey), CO (light turquoise), H_2_ (red), and H_2_O (blue) after pulsing of a C_3_H_8_/Ar = 1:1 mixture over oxidized a) IM_Pt2Ga/Al and b) C_Pt/8GaAl. c) The ratio of t_max_(H_2_)/t_max_(Ar) determined for the oxidized (blue bars) and reduced (red bars) catalysts using the respective responses in a), b) and in Figure S12a,b. d) The rate of propene formation (r(C_3_H_6_)) over the oxidized and reduced catalysts in PDH at 550°C versus the ratio of t_max_(H_2_)/t_max_(Ar). e)–f) The reaction coordinates of H_2_ formation over fully oxidized Ga_2_O_3_ or partially reduced GaO*_x_*.

Mechanistic and kinetic aspects of product formation from propane were further studied using a temporal analysis of products (TAP‐2) reactor operating with sub millisecond resolution.^[^ ^27^, ^28^, ^29^ ^]^ C_3_H_6_, H_2_, CO_x_, and H_2_O were detected after pulsing a C_3_H_8_/Ar = 1/1 mixture over oxidized IM_Pt2Ga/Al and C_Pt/8GaAl at 550 °C (Figure 4a,b). Due to the unsatisfactory quality of the water response, characterized by a high level of noise, its shape and the appearance order were not included in the subsequent discussion. The presence of carbon oxides prove that lattice oxygen of GaO_x_ is able to oxidize propane/propylene. Since C_3_H_8_ was converted into the above products, its response was the narrowest. The response of C_3_H_6_ appears directly after that of C_3_H_8_ while the responses of CO, CO_2_, and H_2_ are shifted to longer times (Figure 4a,b). The delayed appearance of carbon oxides can be explained by the formation of these products through oxidation of primarily formed propylene with the involvement of lattice oxygen. No carbon oxides were observed when propane was pulsed over the reduced catalysts. C_3_H_6_ and H_2_ were the only products (Figure S12a,b).

Comparing the time of the maximal concentration of the H_2_ and C_3_H_6_ responses, one can clearly see in Supplementary Figure S12c that their ratio (*t*_max_(H_2_)/*t*_max_(C_3_H_6_)) is higher in the case of the reduced catalysts in comparison with their oxidized counterparts (about 1 versus about 0.74). This suggests that the reduced catalyst form H_2_ slower. This is also indirectly supported by the ratio of *t*_max_(H_2_)/*t*_max_(Ar) (Figure 4c). The *t*_max_(Ar) stands for mass transport only. It is also worth noting that despite the *t*_max_ values of the H_2_ and C_3_H_6_ responses are close to each other, the formation of H_2_ must be decoupled from the formation of C_3_H_6_ and proceeds at a lower rate. The similar *t*_max_ values of the responses of C_3_H_6_ and H_2_ are because the diffusion coefficient of H_2_ is about 4.6 times higher than that of C_3_H_6_ resulting in faster diffusion of H_2_ through the reactor. The conclusion about the rates of H_2_ and C_3_H_6_ formation is supported by our simple kinetic analysis, which considers the formation of H_2_ and C_3_H_6_ with the same rate (Figure S13 and the respective discussion). Furthermore, we found a correlation between the rate of propene formation and the ratio of *t*_max_(H_2_)/*t*_max_(Ar) as seen in Figure 4d. The higher the ratio, the lower the rate. This correlation also supports our conclusion that hydrogen formation should be the rate‐limiting step in the course of the PDH reaction.

The above conclusion was further supported by density functional theory calculations of H_2_ formation over both fully oxidized Ga_2_O_3_ and partially reduced GaO*_x_*. Specifically, the energy barrier for the recombination of hydrogen atoms derived from GaH^*^ and OH^*^ on the surface of Ga_2_O_3_ is 0.38 eV, which is notably lower than the barrier of 0.93 eV associated with the H*Ga‐GaH* recombination process occurring on partially reduced GaO*_x_* (Figure 4e).

### Catalyst Durability and Benchmarking

**Figure 5:**
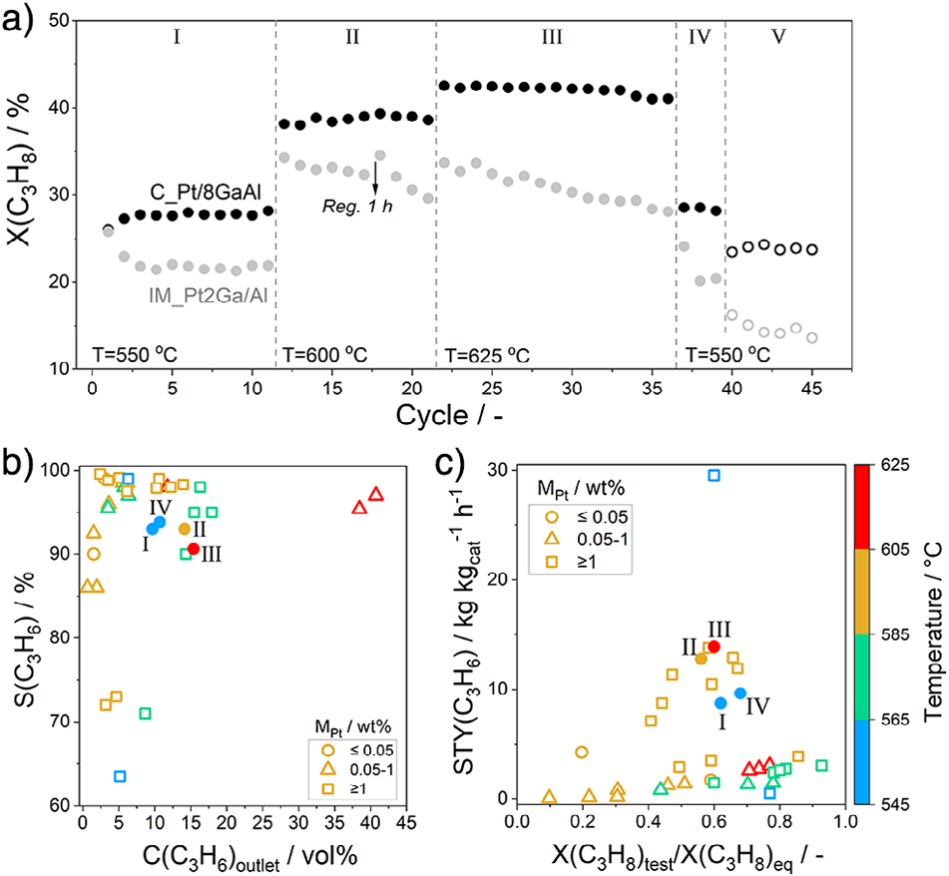
a) Initial propane conversion over C_Pt/8GaAl (black) and IM_Pt2Ga/Al (gray) in a series of 45 PDH/oxidative regeneration cycles under different conditions: C_3_H_8_/N_2_ = 4/6 (solid symbols), C_3_H_8_/H_2_/N_2_ = 4/1/5 (open symbols), *m* = 0.03 g, WHSV(C_3_H_8_) = 37.7 h^−1^. The conversion was determined after 20 min on propane stream in each reaction cycle. b) S(C_3_H_6_) versus the outlet concentration of propylene and c) STY(C_3_H_6_) versus the ratio of the experimentally determined propane conversion to the equilibrium conversion over different PtGa‐based catalysts (Table S1). The datapoints marked with I, II, III and IV were obtained using the results in periods I, II, III, and IV in a), respectively.

To demonstrate the potential of our alternatively developed PtGa‐based catalysts, i.e., C_Pt/8GaAl, we performed a test consisting of a series of PDH/oxidative regeneration cycles at different temperatures (550 °C–625 °C) using industrially relevant feeds with 40 vol% C_3_H_8_. Unless otherwise specified, both the PDH and regeneration lasted for 20 min in each cycle (Figure S14). The whole test was divided into five periods. In the first three periods (I, II, and III), the reaction temperature was 550 °C, 600 °C, and 625 °C, respectively. It then returned to 550 °C in period IV. The temperature remained 550 °C in period V, while the reaction feed was changed from 40 vol% C_3_H_8_ in N_2_ to 40 vol% C_3_H_8_ and 10 vol% H_2_ in N_2_. The C_Pt/8GaAl catalyst could completely restore the initial propane conversion in each cycle of all periods. As expected, the conversion also increased as the reaction temperature increased (Figure 5a). However, the IM_Pt2Ga/Al catalyst failed to restore its activity. The initial propane conversion decreased from 25.8% to 21.4% in the first 4 cycles in period I and then remained stable in cycles 5–11. In period II, the initial propane conversion dropped slightly from 34.3% to 32.3% within cycles 12–17. When the regenerating duration was extended from 20 to 60 min, the activity was totally recovered in cycle 18. Once the regeneration duration returned to 20 min, the conversion dropped significantly from 34.5% to 29.6% within cycles 19–21. Unlike C_Pt/8GaAl, IM_Pt2Ga/Al did not further improve its activity as the temperature increased from 600 °C to 625 °C (period III). When the temperature returned to 550 °C in period IV, the propane conversion over IM_Pt2Ga/Al was 24% in cycle 37 and decreased to 20% in cycle 39. When PDH was performed with co‐fed H_2_ (period IV), the conversion of propane over C_Pt/8GaAl did not significantly drop, while a strong decrease was found for IM_Pt2Ga/Al. This is probably due to the faster formation of H_2_ over the former catalyst.

To derive an insight into the different durability of the C_0.05Pt/8GaAl and IM_0.05Pt2Ga/Al catalysts (Figure 5a), we analyzed both oxidized and reduced samples of these catalysts by scanning transmission electron microscope. Pt species exist as single atoms on the surface of oxidized IM_0.05Pt2Ga/Al but were partially transformed into PtGa particles after catalyst reduction (Figure S15a,b). In contrast, Pt species exist as single atoms on the surface of both oxidized and reduced C_0.05Pt/8GaAl samples (Figure S15c,d). As Pt is required to facilitate the recombination of surface H atoms to make the PDH sites free, the higher durability of the latter catalyst should be related to its ability to keep high dispersion of Pt after a series of PDH/oxidative regeneration cycles.

We also benchmarked the C_Pt/8GaAl catalyst against state‐of‐the‐art PtGa‐based catalysts in terms of propylene selectivity (S(C_3_H_6_)) and space‐time‐yield (STY(C_3_H_6_)) of propylene formation (Figure 5b,c and Table S1). To ensure a proper comparison of the catalysts tested under different conditions, we plotted the S(C_3_H_6_) or STY(C_3_H_6_) values versus the outlet concentration of propylene (Figure 5b) or the ratio of the experimentally determined propane conversion to the corresponding equilibrium conversion (Figure 5c). Most catalysts including the present catalyst showed the selectivity above 90%. The previously reported catalysts have however higher concentration of Pt.

The C_Pt/8GaAl catalyst achieved the initial STY(C_3_H_6_) of 8.7 kg kg_cat_^−1^ h^−1^ at 62% equilibrium propane conversion at 550 °C. As the reaction temperature sequentially increased to 600 °C and 625 °C, the STY(C_3_H_6_) values increased to 12.8 and 13.9 kg kg_cat_^−1^ h^−1^, respectively. These values are higher than those of almost all previously reported PtGa‐containing catalysts. Only Ga^δ+^Pt^0^/SiO_2_ performed superior with STY(C_3_H_6_) of 31.5 kg kg_cat_^−1^ h^−1^ at about 60% equilibrium propane conversion at 550 °C.^[^ ^30^ ^]^ However, the Pt loading of this catalyst is as high as 4.37 wt%, while the Pt loading of the C_Pt/8GaAl catalyst is only 0.05 wt%. When considering the STY(C_3_H_6_) based on Pt amount, the catalyst developed in the present study outperforms all previously reported PtGa‐based catalysts (Figure S16).

## Conclusion

In this study, we demonstrate that in contrast to typically prepared Al_2_O_3_‐supported Pt‐GaO*_x_*‐containing catalysts, the use of a mixed GaAlO*_x_* support for Pt is key to hinder catalyst deactivation with increasing time on propane stream and to ensure durable operation. The best‐performing developed Pt/GaAlO*_x_* catalyst with only 500 ppm Pt outperformed state‐of‐the‐art Pt‐GaO*_x_*‐containing catalysts in terms of space‐time yield of propylene formation at 60%–70% equilibrium conversion and fully recovered its initial performance in a series of PDH/oxidative regeneration cycles in the temperature range 550 °C–625 °C. A combination of H_2_‐TPR, O_2_ titration of reduced catalyst sites after different times on propane stream and in situ UV–vis spectroscopic tests with transient and steady state kinetic studies enabled us to identify the kind of active species and the origins of catalyst deactivation and durability. The recombination of surface hydrogen species formed over GaO_x_ species upon the cleavage of two C─H bonds in propane is the rate‐limiting step in the PDH reaction. This process is accelerated by Pt species and also proceeds more readily on oxidized GaO*_x_* species than on their reduced counterparts. This is the reason for the higher PDH activity of the former species. The reaction‐induced reduction of oxidized GaO*_x_* and agglomeration of Pt species were found to cause catalyst deactivation during propane dehydrogenation and in a series of dehydrogenation/oxidative regeneration cycles. These undesirable transformations are hindered in mixed GaAlO_x_ compared to GaO_x_ species supported on Al_2_O_3_ due to the increased strength of the Ga─O─Al bond.

## Conflict of Interests

The authors declare no conflict of interest.

## Supporting information



Supporting Information

## Data Availability

The data that support the findings of this study are available from the corresponding author upon reasonable request.
