# Supplementary material for: The Role of Reducibility of PtGaO x ‐Based Catalysts for Efficient and Durable Propane Dehydrogenation
Source: Angew Chem Int Ed Engl. 2025 Jun 16;64(31):e202506704. doi: 10.1002/anie.202506704 (PMC12304820; doi:10.1002/anie.202506704)
Supplement: Supplementary file 1 — Supporting Information [file ANIE-64-e202506704-s001.pdf]

Supporting Information
©Wiley-VCH 2021
69451 Weinheim, Germany

The Role of Reducibility of $PtGaO_x$-based Catalysts for Efficient and Durable Propane Dehydrogenation

Kai Wu, Vita A. Kondratenko, Mingxia Zhou, Dmitry E. Doronkin, Stephan Bartling, Qiyang Zhang, Shanlei Han, Xin Jia, Qi Liu, Dong Xiong, Chunming Xu, Guiyuan Jiang*, Dan Zhao*, Uwe Rodemerck, David Linke, and Evgenii V. Kondratenko*

Abstract: Although $PtGaO_x$-containing catalysts are active and selective in the non-oxidative dehydrogenation of propane (PDH) to propylene, they suffer from rapid deactivation and, especially, inability to recover their initial performance in a series of PDH/oxidative regeneration cycles, characteristics that are highly relevant to commercialization. Herein, we identified reducibility of GaO_x as the key descriptor affecting the above catalyst features. Oxidized GaO_x species are more active than reduced GaO_x in the recombination of two H species formed from propane, which is the rate-limiting step in the PDH reaction. This process is further accelerated by Pt. The reduction of GaO_x with time on propane stream leads to catalyst deactivation. Easily reducible GaO_x also tend to form $PtGaO_x$ under PDH conditions, from which Pt atoms present in fresh catalysts cannot be completely recovered during oxidative regeneration, which is detrimental to catalyst durability. Regardless of the reaction atmosphere, Pt single atoms exist on the surface of $PtGaO_x$-containing catalysts with hardly reducible GaO_x. Based on the knowledge derived, we developed a catalyst with 500 ppm Pt on the surface of mixed $GaAlO_x$, which outperforms almost all previous $PtGaO_x$-containing catalysts in terms of space-time yield of propylene formation and shows durable operation under industrially relevant conditions.

DOI: 10.1002/anie.2021XXXXX

Table of Contents

Experimental Procedures 2
Supplementary Figures 5
Supplementary Tables 21
References 24
Author Contributions 24

Experimental Procedures

1.1. Catalyst preparation

An impregnation method was used to prepared two series of catalysts abbreviated as IM_0.05PtxGa/Al (0.2 ≤ x ≤ 2 wt%) and IM_yPt2Ga/Al (0.01 ≤ y ≤ 0.1 wt%). $H_2PtCl_6{\cdot}6H_2O$ (Sigma Aldrich, 99.9%) and $Ga(NO_3)_3{\cdot}xH_2O$ (Merk, 99.0%) were used as precursors. Al_2O_3 (Chempur, acidic) was used as a support. After impregnation, the impregnated precursors of the catalysts were dried at 110 ºC under ambient conditions over night and then calcined at 550 ºC in air for 6 h. The IM_0.05PtxGa/Al catalysts have a fixed Pt content of 0.05wt% but different Ga loadings and the IM_yPt2Ga/Al catalysts have fixed Ga loading of 2 wt% but different contents of Pt.

For the synthesis of the C_Pt/GaAl catalysts, $GaAlO_x$ solid solutions were firstly prepared by a co-precipitation method. $Ga(NO_3)_3{\cdot}xH_2O$, $Al(NO_3)_3{\cdot}9H_2O$ (Chempur, >98%) and $NH_3{\cdot}H_2O$ (ROTH, 25 wt%) were used without any purification. 18 mL of $NH_3{\cdot}H_2O$ (25 wt%) was added dropwise to 250 mL of $Al(NO_3)_3$ and $Ga(NO_3)_3$ mixed solution under continuous stirring. The concentration of Al^{3+} is 0.1 mol L^{-1}. The weight ratio of Ga in $GaAlO_x$ is 2-25 wt%. The formed suspension was stirred at room temperature for an additional 1 h followed by aging at room temperature for 48 h. The solid product was collected after filtration, washed with deionized water, dried at room temperature, and then calcined at 550 ºC in air for 6 h. Then, the obtained solid materials were used as supports and impregnated with Pt. Finally, the catalysts were obtained by calcination at 550 ºC in air for 6 h. The calcined materials were pressed, crushed, and sieved to get particles of 315-710 µm before catalytic tests.

1.2. Characterizations

Pulse titration experiments were carried out with a mixture of 1vol% O_2 in He using an in-house (Leibniz Institut für Katalyse e.V.) developed setup. Pulsing was realized by a six-port valve with a 1 mL loop. Argon was used as a carrier gas. 100 mg of each spent catalyst after different times on propane stream was placed in a quartz fixed-bed continuous-flow reactor between two quartz wool pieces. A layer of 800 mg SiC was placed on top of the catalyst bed. The catalysts were initially heated to 550 °C in a flow of argon. The O_2-containing mixture was pulsed manually with a time interval of 1 min. Pulse stopped when no significant O_2 consumption and CO_2 formation were observed. The atomic mass units (AMUs) of 32 (O_2) and 40 (CO_2) were used for the analysis.

Temperature-programmed reduction experiments with CO (CO-TPR) were carried out in a setup developed in-house (Leibniz Institut für Katalyse e.V.) containing eight individually heated continuous-flow fixed-bed quartz reactors. 100 mg of each catalyst was first calcined in a flow of air at 550 °C for 1 h and then cooled down to 40°C. Hereafter, a flow of 1 vol% CO/Ar (10 mL min^{-1}) was fed to the reactor and the temperature was increased from 40 to 900°C with a heating rate of 10°C min^{-1}. The consumption of CO was detected by an online mass spectrometer (Pfeiffer Vacuum OmniStar GSD 320). AMUs of 28 and 40 were recorded for monitoring CO and Ar.

X-ray photoelectron (XP) spectroscopy measurements were carried out on an ESCALAB 220iXL (Thermo Fisher Scientific) instrument with monochromated Al Kα radiation (E = 1486.6 eV). The electron binding energies were obtained with charge compensation using a flood electron source and referenced to the C 1s core level of carbon at 284.8 eV (C-C and C-H bonds).

X-ray absorption spectra (XANES and EXAFS) at the Ga K absorption edge were recorded at the P65 beamline of PETRA III synchrotron radiation source (DESY, Hamburg) in transmission mode. Higher harmonics were rejected by a pair of Si plane mirrors installed in front of the monochromator. The energy of the X-ray photons was further selected by a Si (111) double-crystal monochromator and the beam size was set by means of slits to 0.4 (vertical) × 2.0 (horizontal) mm^2. For the measurements, W foil was used as a reference foil. The spectra were normalized and the extended X-ray absorption fine structure spectra (EXAFS) background subtracted using the ATHENA program from the IFEFFIT software package[1]. The k^2-weighted EXAFS functions were Fourier transformed (FT) in the k range of 3-12 $Å^{-1}$ and multiplied by a Hanning window with sill size of 1 $Å^{-1}$. The analyzed r range was 1-3 Å. The displayed FT EXAFS spectra were not corrected for the phase shift. S_0^2=0.90 was obtained by fitting the Cu foil spectrum which was measured at the same beamline and used for further fittings.

Time-resolved in-situ UV-Vis experiments were used for analyzing the kinetics of the reduction of the developed oxidized catalysts. The measurements were performed using an AVASPEC fiber optical spectrometer (Avantes) equipped with a DH-2000 deuterium-halogen light source and a CCD array detector. A high-temperature reflective UV visible probe consisting of six radiating fibers and one reading fiber is located inside the furnace, perpendicular to the quartz tube reactor. The catalyst was first calcined in the air at 550 °C for 1 h and then exposed to a flow of 25 vol% H_2/N_2 at the same temperature for 0.5 h. UV-Vis spectra (from 200 to 800 nm) were recorded over time as the reaction progressed. The spectra were converted into the Kubelka-Munk function F(R). For analysis of the reduction kinetics, we reported the relative Kubelka-Munk function $F(R_{rel})$ with respect to the fully oxidized sample. $F(R_{rel})$ was calculated by Eq. S1-2.

$$R_{rel}=\frac{R_{H_2}}{R_{O_2}} \qquad \text{(S1)}$$

$$F(R_{rel})=\frac{(1-R_{rel})^2}{2\times R_{rel}} \qquad \text{(S2)}$$

Transient tests of propane dehydrogenation were performed in a Temporal Analysis of Products (TAP-2) reactor, a transient technique that operates in vacuum with sub-millisecond resolution.[2] 50 mg of each catalyst (IM_Pt2Ga/Al or C_Pt/8GaAl (sieve fraction of 250-450 µm)) was packed between two layers of quartz particles (sieve fraction of 250-355 µm) within the isothermal zone of a tube reactor made of quartz. Prior to the pulse experiments, the catalysts were subjected to two distinct treatment regimes. An oxidative treatment was performed in a flow of O_2 (4 mL min^{-1}) for 30 min at 550°C. The catalyst treated in this way is referred to as oxidized catalyst. The so-called reduced catalyst was additionally exposed to a flow of H_2 (2 mL min^{-1}) and Ar (2 mL min^{-1}) for another 30 min at 550°C after the treatment in O_2. After the treatment, the reactor with the catalyst was evacuated to about 10^{-5} Pa and pulse experiments with a C_3H_8:Ar = 1:1 mixture were performed. C_3H_8 (Linde, 3.5), H_2 (Air Liquide, 5.0), O_2 (Air Liquide, 4.5), and Ar (Air Liquide, 5.0) were used without additional purification. Transient responses associated with the feed components and the reaction products were monitored at the reactor outlet using a quadrupole mass spectrometer (HAL RD 301 Hiden Analytical) at following AMU values: 44 (C_3H_8, CO_2), 42 (C_3H_8, C_3H_6), 41 (C_3H_8, C_3H_6), 29 (C_3H_8), 28 (C_3H_8, C_2H_4, CO_2, CO), 26 (C_3H_8, C_3H_6, C_2H_4), 18 (H_2O), 15 (CH_4), 2 (H_2), and 40 (Ar). For each AMU, pulses were repeated 10 times and averaged to improve the signal-to-noise ratio.

1.3. Catalytic Tests

Catalytic PDH experiments were performed using an in-house (Leibniz Institut für Katalyse e.V.) developed setup with 15 fixed-bed quartz reactors operating in parallel at ambient pressure. Each of the catalysts was loaded into a fixed-bed quartz tubular reactor. Before the tests, the catalysts were heated in a flow of air to 550 °C and then held at this temperature for 1 h, denoted as oxidized materials. The reduced catalysts were obtained from their oxidized counterparts using an additional treatment at the same temperature in a 50 vol% H_2/N_2 mixture for another 1 h. The feed composition for PDH tests was C_3H_8/N_2 = 40:60 or $C_3H_8/H_2/N_2$ = 40:10:50. N_2 was used as diluent and internal standard for considering the reaction-induced changes in the number of moles. The amount of catalysts was varied to obtain different contact times and accordingly different degrees of C_3H_8 conversion. For the determination of the propene formation rate ($r(C_3H_6)$), different catalyst amounts (10-50 mg) and reaction feed flows (40-70 mL min^{-1}) were altered to achieve a propane conversion below 10% at 550 °C. A durability test consisted of 45 PDH/oxidative regeneration cycles with a WHSV(C_3H_8) of 37.7 h^{-1} at different temperatures (550-625 °C). Unless otherwise specified, both PDH and regeneration cycles lasted for 20 min. Air was used in the regeneration cycles. Plug-flow conditions and feed gas preheating were ensured by positioning a layer of silicon carbide on the top of the catalyst bed. An online gas chromatograph (Agilent 7890) was used to quantify the concentration of the feed components and the reaction products. Propane conversion $X(C_3H_8)$, product selectivity S(i), the propylene formation rate $r(C_3H_6)$ and space-time yield of propene STY were calculated according to the following equations (Eq. S3-7):

$$X(C_3H_8)=\frac{\dot{n}^{in}_{C_3H_8}-\dot{n}^{out}_{C_3H_8}\frac{\dot{n}^{in}_{N_2}}{\dot{n}^{out}_{N_2}}}{\dot{n}^{in}_{C_3H_8}} \qquad \text{(S3)}$$

$$S(i)=\frac{v_i}{v_{C_3H_8}}\frac{\dot{n}^{out}_{C_3H_8}}{\dot{n}^{in}_{C_3H_8}-\dot{n}^{out}_{C_3H_8}} \qquad \text{(S4)}$$

$$S(coke)=1-\sum_i S(i) \qquad \text{(S5)}$$

$$r(C_3H_6)=\frac{\dot{n}_{C_3H_6}}{m_{\mathrm{cat}}} \qquad \text{(S6)}$$

$$STY=\frac{\dot{n}_{C_3H_6}\times M_{C_3H_6}\times 60}{1000\times m_{\mathrm{cat}}} \qquad \text{(S7)}$$

where $\dot{n}$ with "*in*" and "*out*" stand for the molar flow of gas phase component (mmol·min^{-1}) at the inlet and outlet, respectively. $v_{C_3H_8}$ and v_i represent the number of carbon atoms in C_3H_8 and product i, respectively. $M_{C_3H_6}$ is the molecular weight of propene (42 g mol^{-1}). n_{Ga} and n_{Pt} stand for concentration of Zn and Pt atoms in the catalysts (mmol g^{-1}). m_{cat} is the mass of catalyst (g).

An apparent turnover frequency (TOF), which is defined as the number of propane molecules converted per Pt or Ga atom per second, was determined according to r(C_3H_6) (Eq. S8-9).

$$TOF_{\mathrm{Ga}} = \frac{r(C_3H_6)}{n_{\mathrm{Ga}} \times 60} \quad \text{(S8)}$$

$$TOF_{\mathrm{Pt}} = \frac{r(C_3H_6)}{n_{\mathrm{Pt}} \times 60} \quad \text{(S9)}$$

where n_{Ga} and n_{Pt} stand for the concentration of Ga and Pt atoms in the catalysts (mmol g^{-1}).

1.4. Theoretical Calculations

Spin-polarized density functional theory (DFT) calculations based on Vienna Ab initio Simulation Package (VASP) were performed.[3] The generalized gradient approximation Perdew-Burke-Ernzerhof (GGA-PBE)[4] functional was employed, including van der Waals corrections. A 3 × 3 × 1 K-point mesh for surface calculations and Γ-point mesh for gas phase calculations were used to approximate reciprocal space integration over Brillouin-zone based on the Monkhorst-Pack scheme.[5] The cutoff energy was set to 600 eV, and self-consistent iterations were converged to 1×10^{-5} eV. Geometry optimizations were converged until the force and energy reached 0.02 eV/Å and 10^{-5} eV, respectively.

The Combined Image Nudged Elastic Band (CI-NEB)[6] and dimer[7] calculations were used to search the transition state (TS) structures and to compute the TS energies. Vibrational frequencies of TS were calculated to confirm the presence of a single imaginary frequency of each TSs. The binding energy (BE) of reaction intermediates with the catalyst surface, reaction energy (ΔE) and energy barrier (E_a) for an elementary step were calculated using Eqs. S10-12, respectively.

$$BE = E_{A*} - E_{*} - E_{A} \quad \text{(S10)}$$

$$\Delta E = E_{FS} - E_{IS} \quad \text{(S11)}$$

$$E_a = E_{TS} - E_{IS} \quad \text{(S12)}$$

The E_{A*} is the total energy of the adsorbate 'A' adsorbed on the surface, E_{*} is the total energy of a pristine surface without any adsorbate, and E_A is the total energy of free adsorbate 'A' in gas phase. The E_{FS}, E_{IS} and E_{TS} are the total energy of the final state, initial state, and TS from DFT calculations.

Supplementary Figures

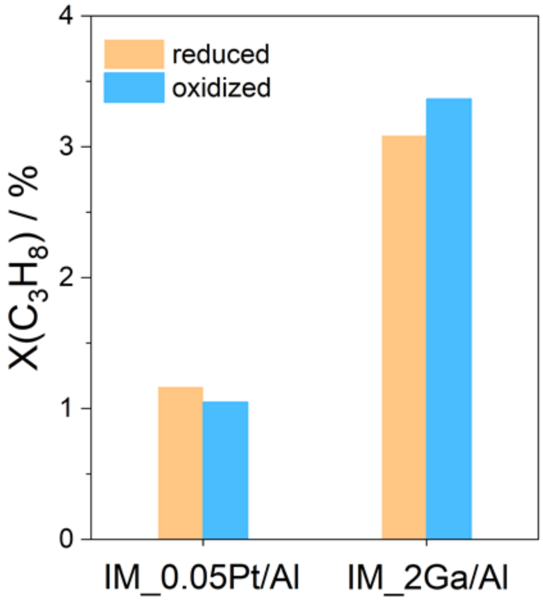

Figure S1. Propane conversion over IM_0.05Pt/Al and IM_2Ga/Al at 550°C using a feed C_3H_8/N_2 = 2/3 at WHSV(C_3H_8) of 37.7 h^{-1}.

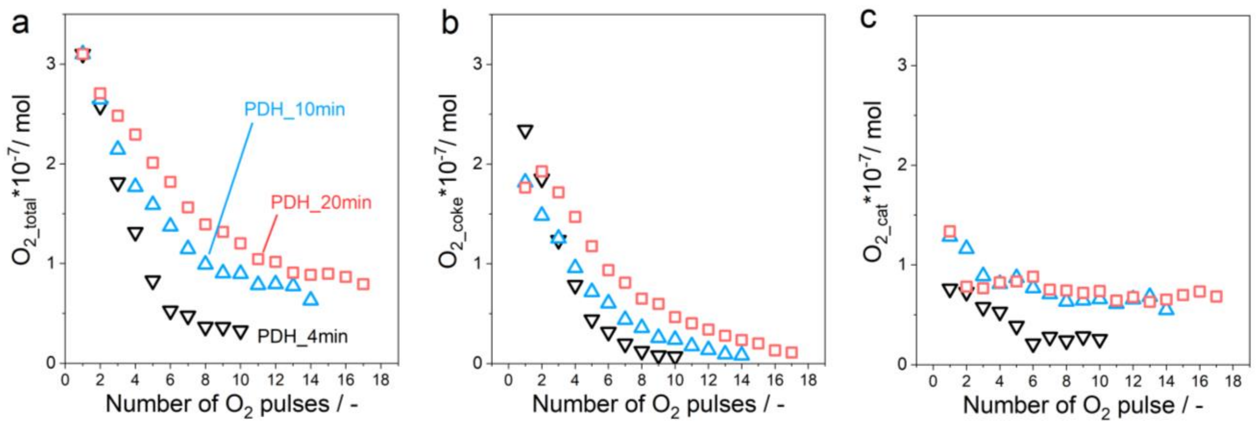

Figure S2. (a) The total amount of O_2 consumed (O_2_total), the amount of O_2 consumed for (b) CO_2 formation (O_2_coke) or (c) reoxidation of spent catalysts (O_2_total) after PDH of 4 min (black triangle), 10 min (blue inverted triangle) and 20 min (orange square).

The amount of O_2 consumed for coke oxidation (O_2_coke) was assumed to be equal to the amount of CO_2 generated. The amount of O_2 consumed (O_2_cat) for reoxidation of reduced GaO_x species is equal to the difference between O_2_total and O_2_coke.

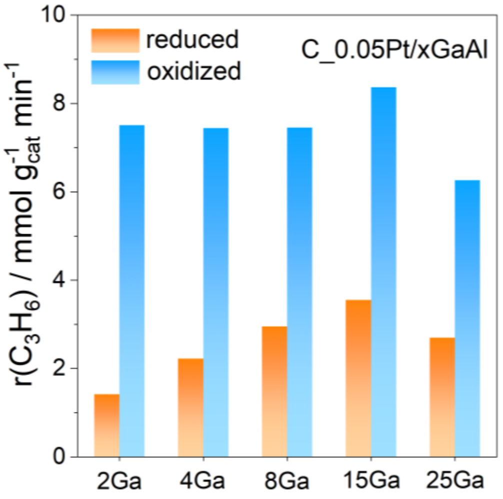

Figure S3. Propylene formation rate (r(C_3H_6)) versus Ga loading over the C_0.05Pt/xGaAl catalysts at 550°C using a feed C_3H_8/N_2 =2/3 at WHSV(C_3H_8) of 37.7 h^{-1}.

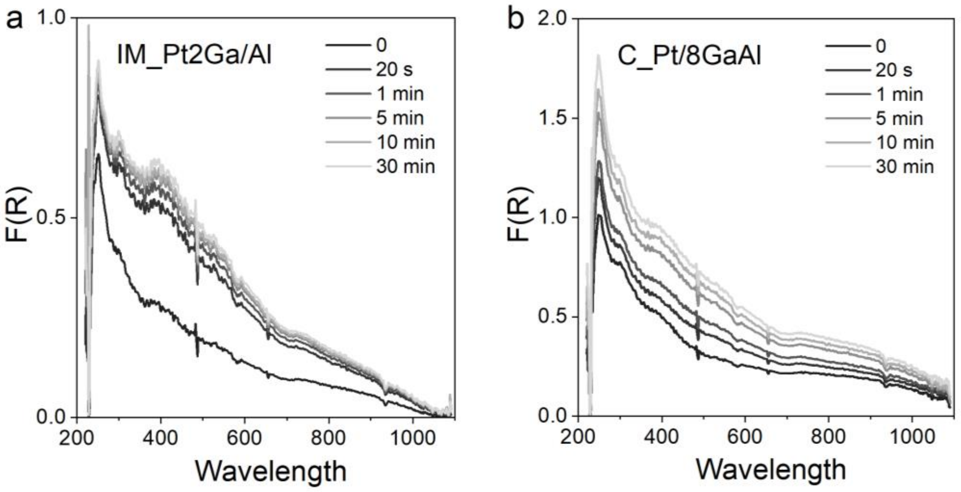

Figure S4. UV-vis spectra (F(R)) of (a) IM_Pt/2Ga/Al and (b) C_Pt/2GaAl at 550 °C after different times on H_2 stream (25 vol% H_2 in N_2).

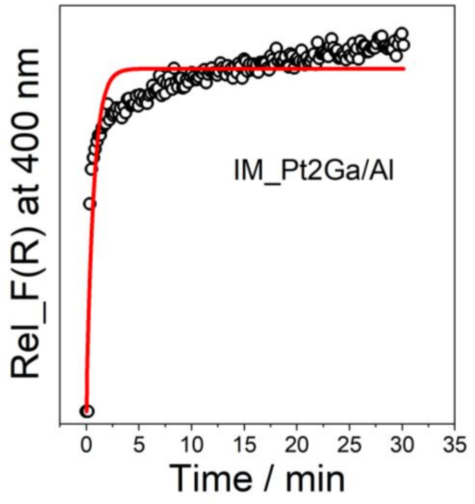

Figure S5. Temporal changes in the Kubelka-Munk function at 400 nm (open black circles) during reduction of fully oxidized IM_Pt2Ga/Al by H_2 (25 vol% H_2/N_2) at 550 °C and the fit (red solid line) according to the 1-site model (equation 1 in the main text).

Time-resolved in-situ UV-Vis spectroscopy was applied to monitor the reduction kinetics of oxidized GaO_x species. Since the intensity of absorption bands at around 400 nm increased with rising time on H_2 stream (Figure S4), we consider the relative Kubelka-Munk function (F(Rrel)) at 400 nm to analyze the reduction kinetics. According to a simple kinetic model, H_2 reacts with an oxidized GaO_x species yielding a reduced GaO_x species as given in equation S13:

$$\mathrm{H_2} + \mathrm{GaO}_{x_{ox}} \xrightarrow{k_1} \mathrm{GaO}_{x_{red}} + \mathrm{H_2O} \qquad \text{(S13)}$$

According to previous studies[8], the mechanistic scheme of above model should be related to the formation rate of reduced species, as described in Eq. S14:

$$\frac{\mathrm{d}C(\mathrm{GaO}_{x_{red}})}{\mathrm{d}t} = k_1 \times p(\mathrm{H_2}) \times C\left(\mathrm{GaO}_{x_{ox}}\right) \qquad \text{(S14)}$$

Integrating Eq. (S15) results in the following expression:

$$C\left(\mathrm{GaO}_{x_{red}}\right) = C^0\left(\mathrm{GaO}_{x_{ox}}\right) \times \left(1\text{-}e^{-k_1^{ap} t}\right) \qquad \text{(S15)}$$

where k_1^{ap} is a product of the reaction constant k_1 of reduction of GaO_x species by H_2 and the partial pressure of H_2. $C\left(\mathrm{GaO}_{x_{red}}\right)$ is the concentration of reduced GaO_x species, $C\left(\mathrm{GaO}_{x_{ox}}\right)$ and $C^0\left(\mathrm{GaO}_{x_{ox}}\right)$ are the concentrations of oxidized GaO_x species in UV-vis beam at t min and 0 min, respectively.

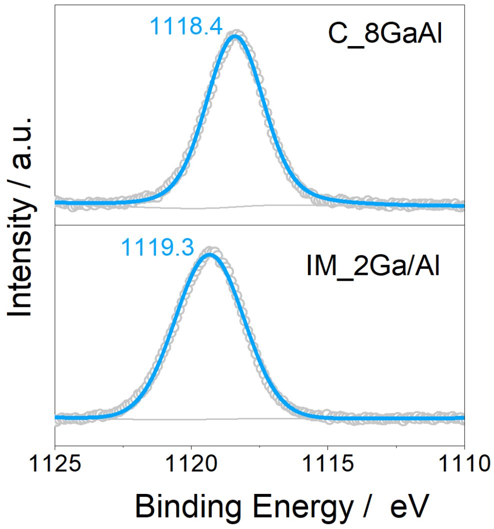

Figure S6. The Ga2p$_{3/2}$ XP spectra of C_8GaAl and IM_2Ga/Al.

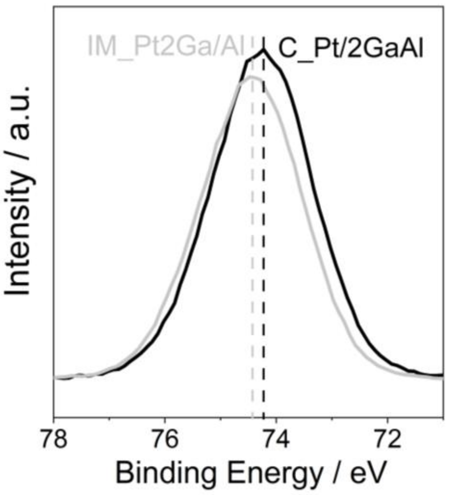

Figure S7. The Al2p$_{3/2}$ XP spectra of C_Pt/2GaAl and IM_Pt/2Ga/Al.

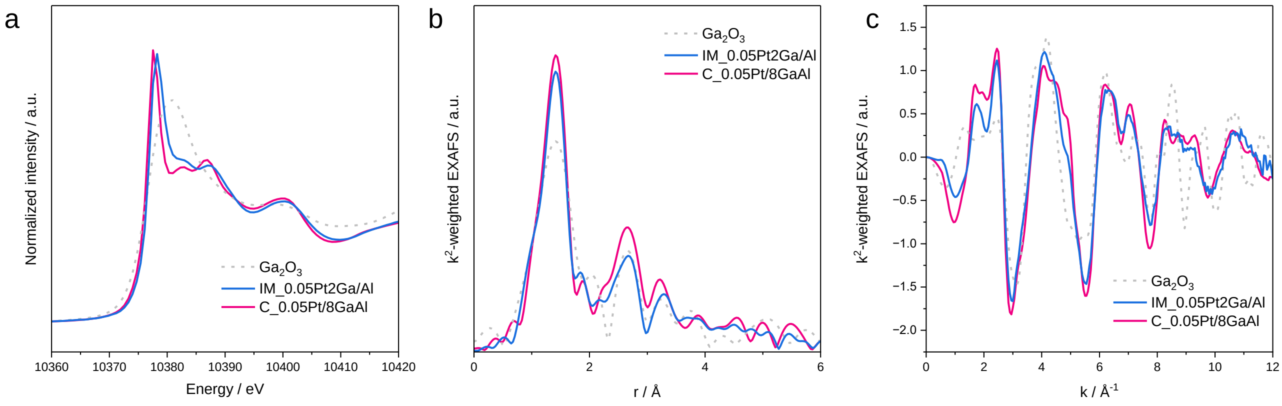

Figure S8. (a) XANES, (b) EXAFS spectra and (c) k^2-weighted EXAFS functions (extracted fine structure in k-space) at the Ga K edge of the IM_0.05Pt2Ga/Al and C_0.05Pt8GaAl catalysts and the Ga_2O_3 reference material. The spectrum of Ga_2O_3 is from the IXAS XAFS database with a number of 521 (http://ixs.iit.edu/database/data/Farrel_Lytle_data/RAW/Ga/index.html).

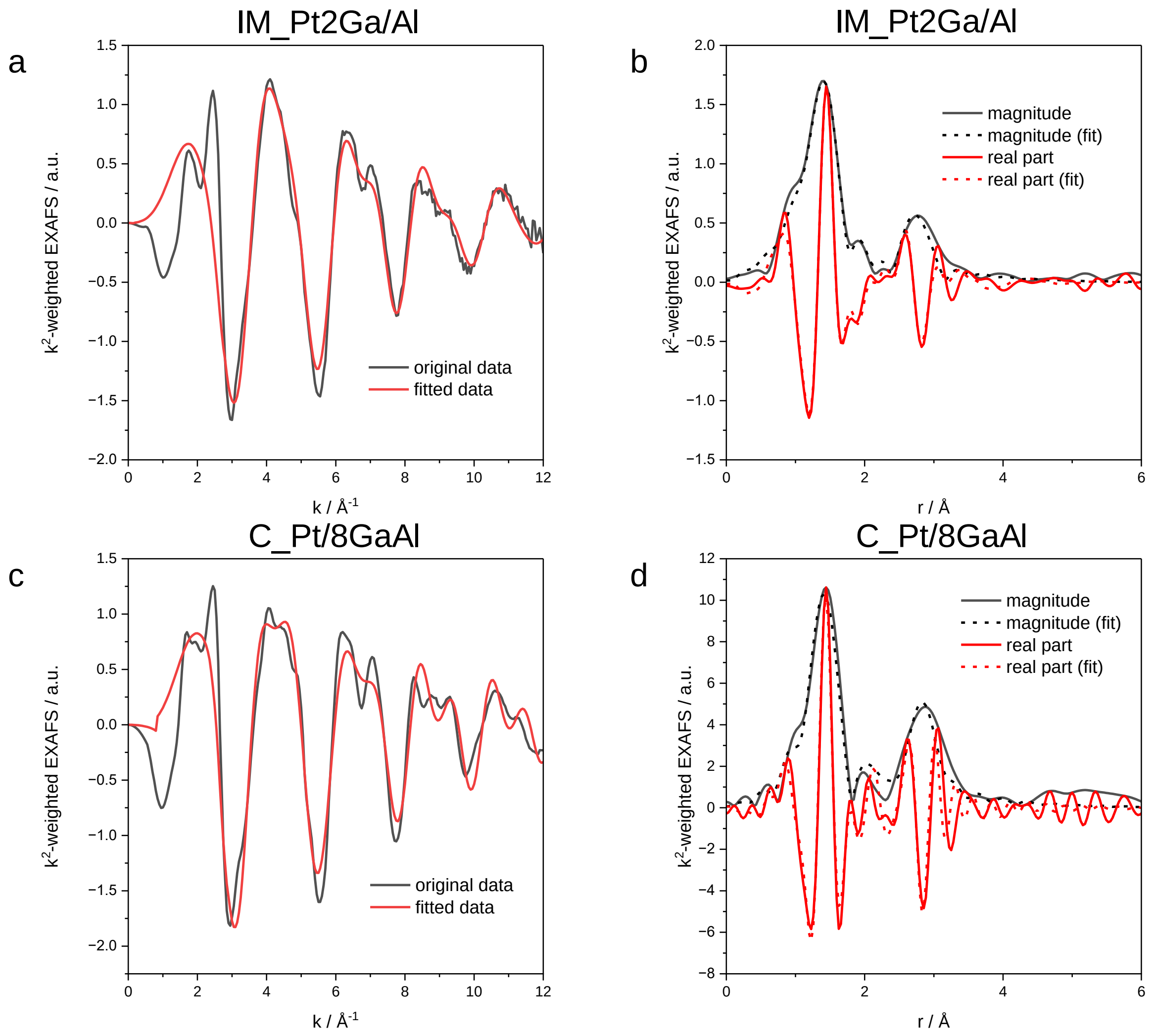

Figure S9. The EXAFS fits for IM_0.05Pt2Ga/Al and C_0.05Pt/8GaAl sample in (a, c) k and (b, d) r space.

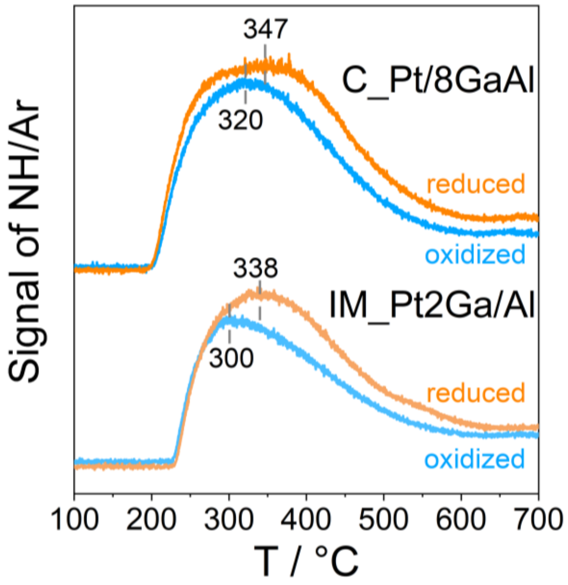

Figure S10. NH_3-TPD profiles of differently treated IM_Pt2Ga/Al and C_Pt/8GaAl

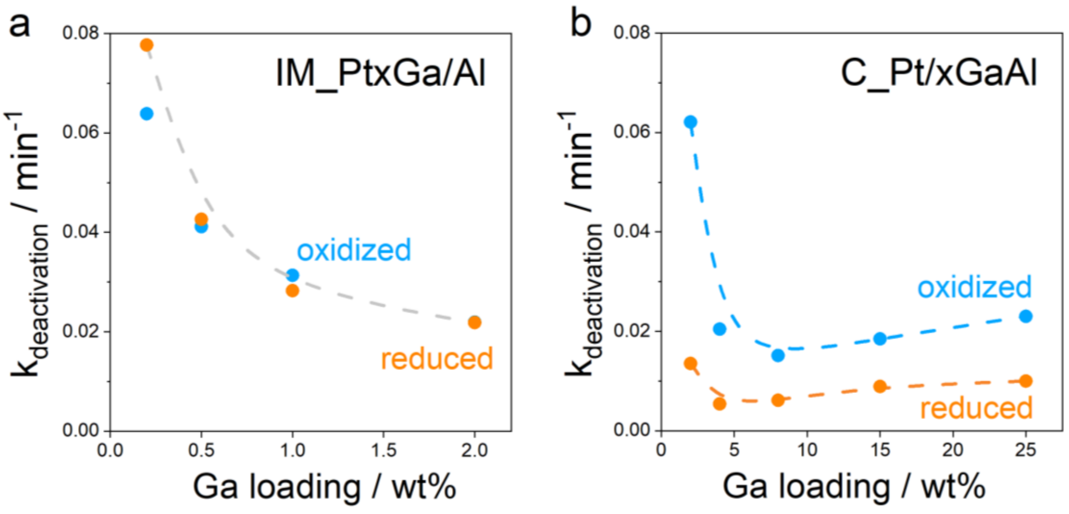

Figure S11. The deactivation rate constant of differently treated (a) IM_PtxGa/Al and (b) C_Pt/xGaAl versus Ga loading.

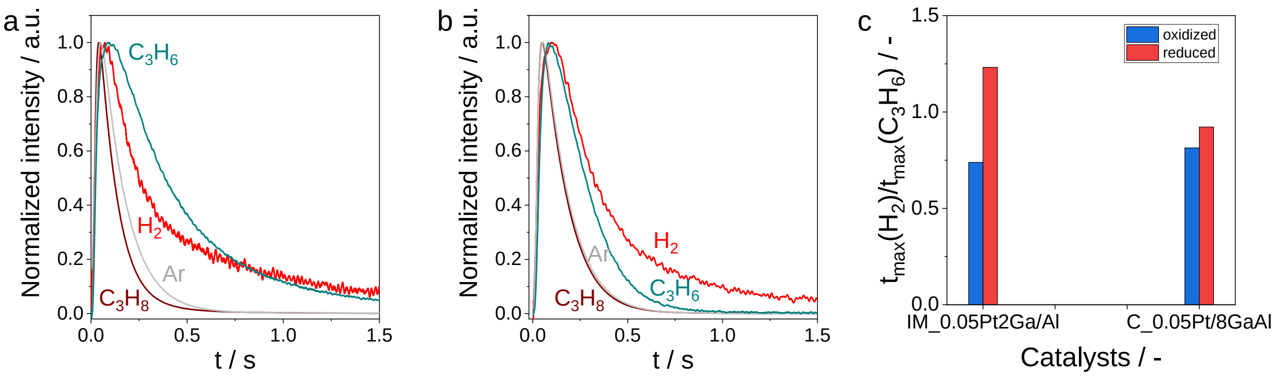

Figure S12. Height-normalized transient responses of Ar, C_3H_8, C_3H_6 and H_2 after pulsing of a C_3H_8/Ar = 1:1 mixture over (a, b) reduced C_0.05Pt/8GaAl and IM_0.05Pt2Ga/Al at 550°C. (c) The ratio of $t_{max}(H_2)/t_{max}(C_3H_6)$ determined from the experimental responses in (a, c) and in Figure 4a,b in the main manuscript.

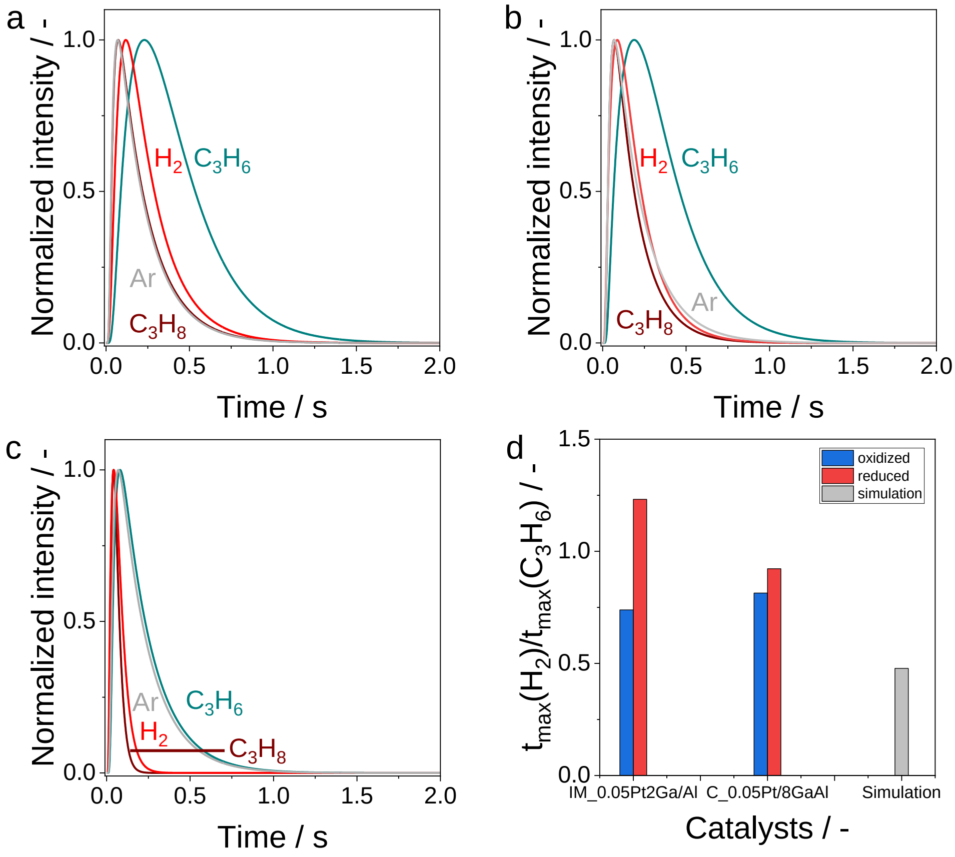

Figure S13. Simulated height-normalized responses of C_3H_8, C_3H_6 and H_2 using the below model. The Ar response was modelled assuming Knudsen diffusion. The k_1 and k_2 values are 5 and 50 s-1 in (a), 50 and 500 s^{-1} in (b) and 5000 and 500 s^{-1} in (c). The catalyst zone is 3 mm, while the total reactor length is 40 mm. The reaction temperature was 550°C. The pulse size of C_3H_8 was 3.5e14 molecules. (d) The ratio of experimental $t_{max}(H_2)/t_{max}(C_3H_6)$ values are from **Figure S12**, while the theoretical value determined from the responses in (b).

Using the model of propane dehydrogenation in two steps: (i) $C_3H_8 + x \xrightarrow{k_1} x\text{-}C_3H_8$ and (ii) $x\text{-}C_3H_8 \xrightarrow{k_2} x + H_2 + C_3H_6$, i.e., C_3H_6 and H_2 are simultaneously formed with the same reaction rate, we simulated the responses of C_3H_8, C_3H_6 and H_2 in a three-zone reactor used in our experiments. The catalyst is located in the isothermal zone between two zones of quartz particles of the same size as the catalyst. The catalyst zone is 3 mm, while the total reactor length is 40 mm. The reaction temperature (in the catalyst zone) was 550°C. The pulse size of C_3H_8 was 3.5e14 molecules. First, we used the rate constants of k_1 and k_2 of 5 and 50 s^{-1}, respectively. The obtained responses as well as the response of Ar (inert standard) are shown in **Figure S13a**. The ratio of $t_{max}(H_2)/t_{max}(C_3H_6)$ in this simulation was 0.51. To increase C_3H_8 conversion (about 3.3% in the former case), we simulated the responses of C_3H_8, C_3H_6 and H_2 using the k_1 and k_2 values of 50 and 500 s^{-1} or 5000 and 500 s^{-1} (**Figure S13b,c**). The obtained corresponding $t_{max}(H_2)/t_{max}(C_3H_6)$ values are about 0.47 and 0.54 at propane conversion of 25 and 97%.

We also calculated the $t_{max}(H_2)/t_{max}(C_3H_6)$ values from the experimental responses of H_2 and C_3H_6 shown in **Figure S12a,b and Figure 4a,b in the main manuscript**. The obtained results are given in **Figure S13d**. It is obvious that the experimental values are higher than the theoretical one. This is an indication for a slower formation rate of H_2 in comparison with C_3H_6.

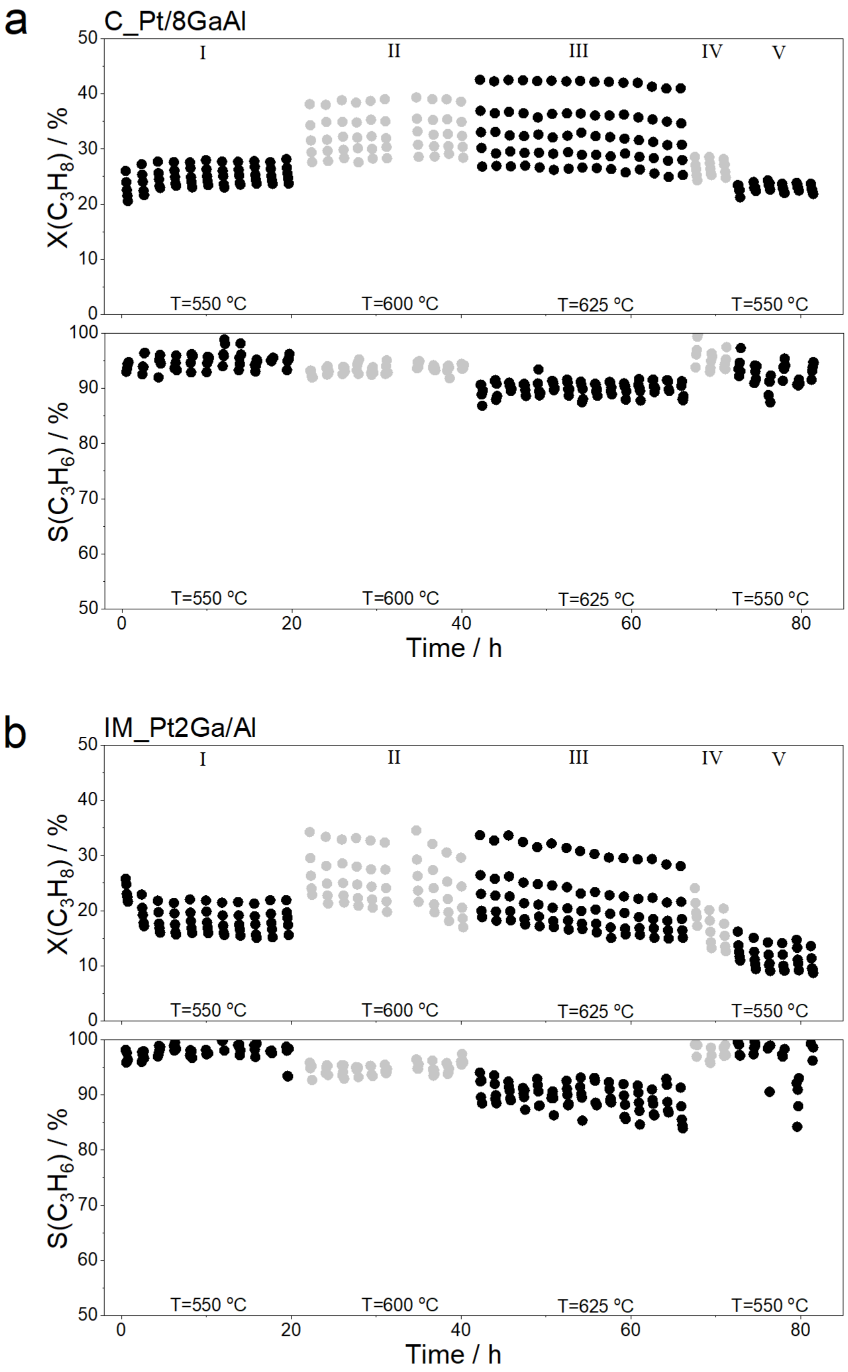

Figure S14. The propane conversion and selectivity to propylene over C_Pt/8GaAl (a) and IM_Pt2Ga/Al (b) within 81 hours under different conditions: (I) 550°C, C_3H_8/N_2 = 2/3, WHSV(C_3H_8) = 37.7 h^{-1}; (II) 600°C, C_3H_8/N_2 = 2/3, WHSV(C_3H_8) = 37.7 h^{-1}; (III) 625°C, C_3H_8/N_2 = 2/3, WHSV(C_3H_8) = 37.7 h^{-1}; (IV) 550°C, C_3H_8/N_2 = 2/3, WHSV(C_3H_8) = 37.7 h^{-1}; (V) $C_3H_8/H_2/N_2$ = 4/1/5, WHSV(C_3H_8) = 37.7 h^{-1}.

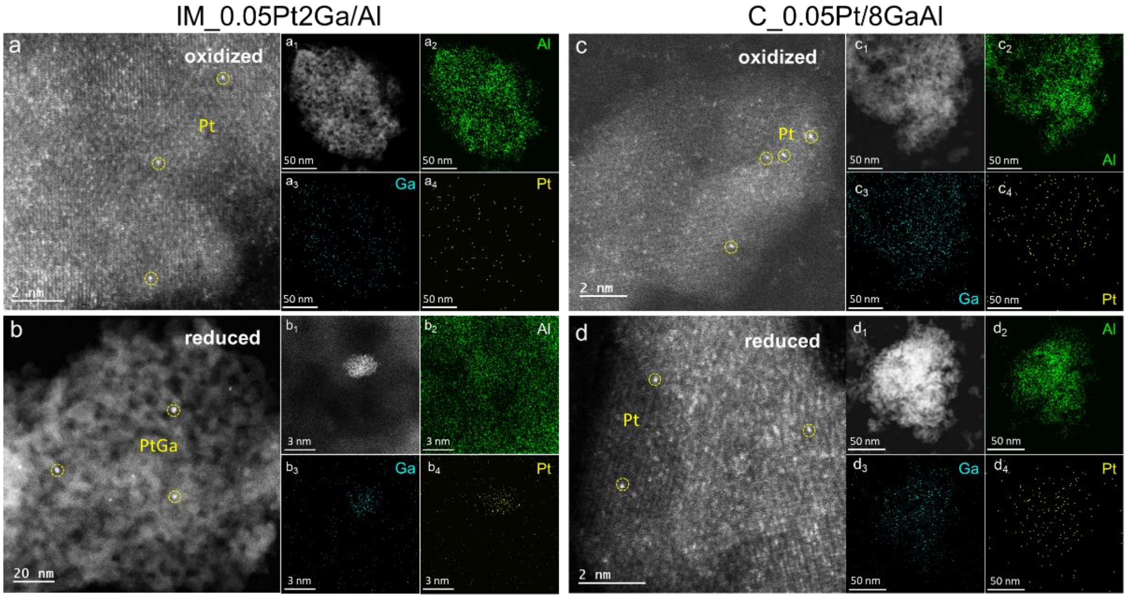

Figure S15. (a, b, c, d) AC-STEM images of oxidized and reduced IM_0.05Pt2Ga/Al and C_0.05Pt/8GaAl. (a_1, b_1, c_1, d_1) HAADF-STEM images of oxidized and reduced IM_0.05Pt2Ga/Al and C_0.05Pt/8GaAl. (a_2-a_4, b_2-b_4, c_2-c_4, d_2-d_4) EDS mapping results for Al, Ga, Pt of oxidized and reduced IM_0.05Pt2Ga/Al and C_0.05Pt/8GaAl.

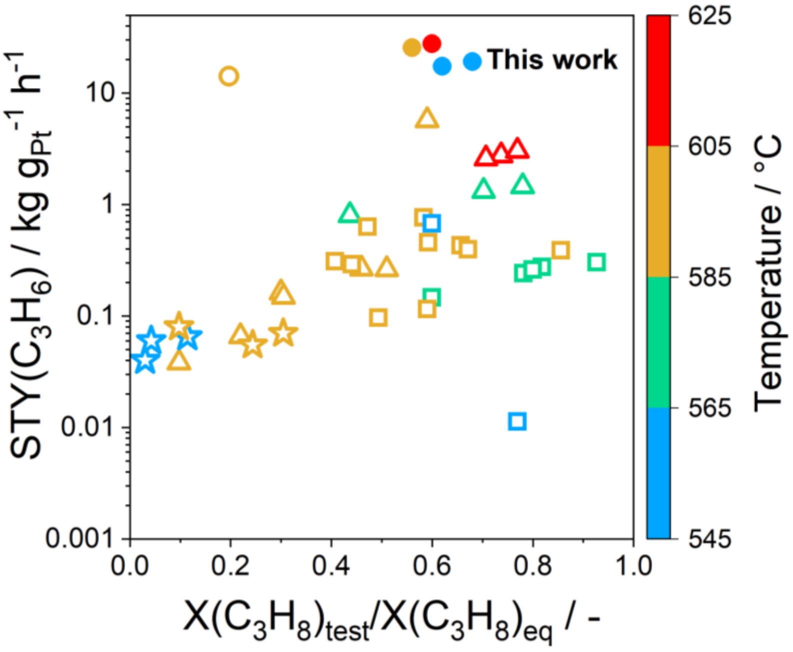

Figure S16. STY(C_3H_6) related to the amount of Pt versus the ratio of the experimentally determined propane conversion to the equilibrium conversion. Details are provided in Table S1.

Supplementary Tables

Table S1. PDH performance of PtGa-based catalysts previously reported in literature and from the present study.

Catalysts	Pt / wt%	Ga / wt%	Feed composition [a]	T / °C	WHSV / h^{-1}	$X(C_3H_8)_{eq}$ / %	$X(C_3H_8)$ / %	$S(C_3H_6)$ / %	X_{test}/X_{eq} / -	$STY(C_3H_6)$ / kg kg_{cat}^{-1} h^{-1}	$STY(C_3H_6)$ [b] / kg g_{Pt}^{-1} h^{-1}	Ref
PtGa@S-1	0.47	0.56	$C_3H_8/H_2/N_2$=10:10:20	600	5.9	49.2	22.5	98.5	0.46	1.25	0.26	[9]
Pt/Ga-S-1	0.5	0.56	$C_3H_8/H_2/N_2$=10:10:20	600	5.9	49.2	15	96	0.30	0.81	0.16	[9]
PtGa/S-1	0.53	0.61	$C_3H_8/H_2/N_2$=10:10:20	600	5.9	49.2	25	98.5	0.51	1.39	0.26	[9]
$Ga^{\delta+}Pt^0/SiO_2$	4.37	1.55	C_3H_8/Ar=10:40	550	98	53	31.9	99	0.60	29.54	0.68	[10]
$Ga^{\delta+}Pt^0/SiO_2$	4.37	1.55	C_3H_8/Ar=2:8	550	2	53	40.7	63.5	0.77	0.49	0.01	[10]
Pt3GaK	0.1	3	C_3H_8=9	620	7.07	57	42	97	0.74	2.75	2.75	[11]
Pt3Ga	0.1	3	C_3H_8=9	620	7.07	57	40.3	95.4	0.71	2.59	2.59	[11]
PtGa/Hd-Al_2O_3	0.1	5	C_3H_8/N_2=2:18	580	2.4	84	65.5	97	0.78	1.46	14.55	[12]
PtGa/Sg-Al_2O_3	0.1	5	C_3H_8/N_2=2:18	580	2.4	84	59	98	0.70	1.32	13.25	[12]
PtGa/C-Al_2O_3	0.1	5	C_3H_8/N_2=2:18	580	2.4	84	36.7	95.5	0.44	0.80	0.80	[12]
PtGa/MgAlO _1redox	2.3	0.2	C_3H_8/H_2/Ar=40:40:120	600	47	54	22	72	0.41	7.11	0.31	[13]
PtGa/MgAlO _ 5redox	2.3	0.2	C_3H_8/H_2/Ar=40:40:120	600	47	54	32	73	0.59	10.48	0.45	[13]
Pt/MgGaAlO _1redox	1.8	1.1	C_3H_8/H_2/Ar=40:40:120	600	47	54	25.5	99.1	0.47	11.34	0.63	[13]
Pt/MgGaAlO _5redox	1.8	1.1	C_3H_8/H_2/Ar=40:40:120	600	47	54	31.5	97.5	0.58	13.78	0.77	[13]
$Ga_{49}Pt/Al_2O_3$	0.12	2.11	C_3H_8/He=8.9:89	550	0.87	65	7.5	97	0.11	0.06	0.065	[14]
$Ga_{49}Pt/Al_2O_3$	0.12	2.11	C_3H_8/He=8.9:89	600	0.87	82	25	87	0.30	0.18	0.07	[14]
$Ga_{48}Pt/SiO_2$	0.21	3.63	C_3H_8/He=8.9:89	550	0.87	65	2	97	0.03	0.02	0.039	[14]

Catalysts	Pt / wt%	Ga / wt%	Feed composition [a]	T / °C	WHSV / h^{-1}	$X(C_3H_8)_{eq}$ / %	$X(C_3H_8)$ / %	$S(C_3H_6)$ / %	X_{test}/X_{eq} / -	$STY(C_3H_6)$ / kg kg_{cat}^{-1} h^{-1}	$STY(C_3H_6)$ [b] / kg g_{Pt}^{-1} h^{-1}	Ref
$Ga_{48}Pt/SiO_2$	0.21	3.63	C_3H_8/He=8.9:89	600	0.87	82	18	92.5	0.22	0.14	0.055	[14]
$Ga_{41}Pt/SiC$	0.15	2.21	C_3H_8/He=8.9:89	550	0.87	65	2.8	98	0.04	0.02	0.06	[14]
$Ga_{41}Pt/SiC$	0.15	2.21	C_3H_8/He=8.9:89	600	0.87	82	8	86	0.10	0.06	0.08	[14]
Pt3Ga/CeAl	1	3	$C_3H_8/H_2/N_2$=13:13:24	600	10	48	41.1	99	0.86	3.88	0.39	[15]
2Ce-Pt/GaAl	0.1	3	C_3H_8/N_2=30:120	620	5.4	78	60	98	0.77	3.03	3.03	[16]
Pt-GaO_x	0.03	3	$C_3H_8/H_2/N_2$=7:7:36	600	41.25	61	12	90	0.20	4.25	14.17	[17]
Pt-GaO_x	0.03	3	$C_3H_8/H_2/N_2$=7:7:36	550	8.25	37	22	99	0.59	1.72	5.72	[17]
Pt/Ga-SBA15	1	1	C_3H_8/N_2=5.55:9.45	580	6.54	55	51	95	0.93	3.02	0.30	[18]
Pt/Ga-MCM48	1	1	C_3H_8/N_2=5.55:9.45	580	6.54	55	45	98	0.82	2.75	0.27	[18]
Pt/Ga-MFI	1	1	C_3H_8/N_2=5.55:9.45	580	6.54	55	43	90	0.78	2.42	0.24	[18]
Pt/Ga-KIT6	1	1	C_3H_8/N_2=5.55:9.45	580	6.54	55	33	71	0.60	1.46	0.15	[18]
Pt/Ga-SiO_2	1	1	C_3H_8/N_2=5.55:9.45	580	6.54	55	44	95	0.80	2.61	0.26	[18]
PtGa-Pb/SiO_2	3	1	C_3H_8/H_2/He=3.9:5:40	600	30.6	68	30	99.6	0.44	8.73	0.29	[19]
PtGa/SiO_2	3	1	C_3H_8/H_2/He=3.9:5:40	600	30.6	68	44.7	98.8	0.66	12.90	0.43	[19]
PtGa-Ca	3	3	C_3H_8/He=2.5:5	600	29.5	64	43	98.3	0.67	11.90	0.40	[20]
PtGa-Ca-Pb	3	3	C_3H_8/He=2.5:5	600	9.8	64	37.8	98	0.59	3.47	0.11	[20]
PtGa-Pb	3	3	C_3H_8/He=2.5:5	600	9.8	64	31.6	97.9	0.49	2.89	0.10	[20]
C_Pt/8GaAl	0.05	8	C_3H_8/N_2=9.6:14.4	550	37.7	42	26.0	93.0	0.62	8.70	17.40	This work
C_Pt/8GaAl	0.05	8	C_3H_8/N_2=9.6:14.4	600	37.7	62	38.1	93.2	0.61	12.78	25.56	
C_Pt/8GaAl	0.05	8	C_3H_8/N_2=9.6:14.4	625	37.7	71	42.5	90.6	0.60	13.86	27.71	
C_Pt/8GaAl	0.05	8	C_3H_8/N_2=9.6:14.4	550	37.7	42	28.5	93.8	0.68	9.62	19.24	

a: The ratio of flow velocity (ml min^{-1});

b: Space time yield of propene based on the mass of Pt.

Table S2. EXAFS fitting results at the Ga K edge.

Sample	Shell	N[a]	R(Å)[b]	σ^2(Å^2)[c]	ΔE_0 (eV)[d]	R factor
IM_0.05Pt2Ga/Al	Ga-O	4.1±0.4	1.84±0.01			
	Ga-O-Ga	0.6±0.4	2.98±0.03	0.0043±0.0011	0.1±1.2	0.0058
	Ga-O-Al	2.3±0.6	3.30±0.03			
C_0.05Pt8GaAl	Ga-O	4.1±0.3	1.85±0.01			
	Ga-O-Ga	3.6±2.0	3.36±0.02	0.0043*	2.5±1.9	0.0127
	Ga-O-Al	6.5±1.8	3.39±0.04			

Note: * means fixed parameter during fitting. S_0^2 was fixed to 0.90 by fitting a known crystal Cu foil which was measured at the same beamline.

References

[1] B. Ravel, M. Newville, *Journal of synchrotron radiation* **2005**, *12*, 537-541.
[2] a) E. V. Kondratenko, J. Perez-Ramirez, *The TAP reactor in catalysis: recent advances in theory and practice*, Elsevier, **2007**; b) K. Morgan, N. Maguire, R. Fushimi, J. Gleaves, A. Goguet, M. Harold, E. V. Kondratenko, U. Menon, Y. Schuurman, G. Yablonsky, *Catal. Sci. Technol.* **2017**, *7*, 2416-2439; c) J. T. Gleaves, G. S. Yablonskii, P. Phanawadee, Y. Schuurman, *Appl. Catal. A: Gen.* **1997**, *160*, 55-88.
[3] a) G. Kresse, J. Hafner, *Physical Review B* **1994**, *49*, 14251; b) G. Kresse, J. Furthmüller, *Computational materials science* **1996**, *6*, 15-50.
[4] J. P. Perdew, K. Burke, M. Ernzerhof, *Physical review letters* **1996**, *77*, 3865.
[5] a) M. de Montigny, L. Marleau, *Physical Review D* **1989**, *40*, 3616; b) H. J. Monkhorst, J. D. Pack, *Physical review B* **1976**, *13*, 5188; c) J. D. Pack, H. J. Monkhorst, *Physical Review B* **1977**, *16*, 1748.
[6] G. Henkelman, B. P. Uberuaga, H. Jónsson, *The Journal of chemical physics* **2000**, *113*, 9901-9904.
[7] G. Henkelman, H. Jónsson, *The Journal of chemical physics* **1999**, *111*, 7010-7022.
[8] O. Ovsitser, M. Cherian, A. Brückner, E. V. Kondratenko, *J. Catal.* **2009**, *265*, 8-18.
[9] B. Zhang, L. Zheng, Z. Zhai, G. Li, G. Liu, *ACS Applied Materials & Interfaces* **2021**, *13*, 16259-16266.
[10] K. Searles, K. W. Chan, J. A. Mendes Burak, D. Zemlyanov, O. Safonova, C. Copéret, *J. Am. Chem. Soc.* **2018**, *140*, 11674-11679.
[11] J. J. Sattler, I. D. Gonzalez - Jimenez, L. Luo, B. A. Stears, A. Malek, D. G. Barton, B. A. Kilos, M. P. Kaminsky, T. W. Verhoeven, E. J. Koers, *Angew. Chem. Int. Ed.* **2014**, *126*, 9405-9410.
[12] Q. Yu, T. Yu, H. Chen, G. Fang, X. Pan, X. Bao, *J. Energy. Chem.* **2020**, *41*, 93-99.
[13] N. V. Srinath, A. Longo, H. Poelman, R. K. Ramachandran, J.-Y. Feng, J. Dendooven, M.-F. Reyniers, V. V. Galvita, *ACS Catal.* **2021**, *11*, 11320-11335.
[14] N. Raman, M. Wolf, M. Heller, N. Heene-Würl, N. Taccardi, M. Haumann, P. Felfer, P. Wasserscheid, *ACS Catal.* **2021**, *11*, 13423-13433.
[15] T. Wang, F. Jiang, G. Liu, L. Zeng, Z. j. Zhao, J. Gong, *AIChE Journal* **2016**, *62*, 4365-4376.
[16] H. C. Kwon, Y. Park, J. Y. Park, R. Ryoo, H. Shin, M. Choi, *ACS Catal.* **2021**, *11*, 10767-10777.
[17] T. Zhang, C. Pei, G. Sun, S. Chen, Z. J. Zhao, S. Sun, Z. Lu, Y. Xu, J. Gong, *Angew. Chem. Int. Ed.* **2022**, *134*, e202201453.
[18] X. Li, P. Rui, W. Huang, X. Yao, Y. Ye, T. Ye, D. J. Morgan, J. H. Carter, *Catal. Lett.* **2024**, *154*, 634-642.
[19] Y. Nakaya, J. Hirayama, S. Yamazoe, K.-i. Shimizu, S. Furukawa, *Nat. Commun.* **2020**, *11*, 2838.
[20] Y. Nakaya, F. Xing, H. Ham, K. i. Shimizu, S. Furukawa, *Angew. Chem. Int. Ed.* **2021**, *60*, 19715-19719.

Author Contributions

E.V.K. conceived and led all stages of this research. E.V.K. and G.J. supervised and coordinated the project. K.W. and Z. D. prepared and characterized the catalysts and carried out the catalytic tests. D.Z. and D.E.D performed XAS measurements and analyzed the data. V.A.K. performed TAP experiments and analyzed the data. M. Z. and X. J. designed and performed the DFT calculations. S. B. performed XPS measurements and analyzed the data. K.W., Q.L., and D.X. performed in-situ IR measurements and analyzed the data. K.W. and Q.Y.Z. analyzed the data of AC-STEM measurements. K.W. and E.V.K. wrote the first draft of the manuscript. All the authors discussed the results and improved the manuscript.
